# NS2 proteases from hepatitis C virus and related hepaciviruses share composite active sites and previously unrecognized intrinsic proteolytic activities

**DOI:** 10.1371/journal.ppat.1006863

**Published:** 2018-02-07

**Authors:** Célia Boukadida, Matthieu Fritz, Brigitte Blumen, Marie-Laure Fogeron, François Penin, Annette Martin

**Affiliations:** 1 Institut Pasteur, Unité de Génétique Moléculaire des Virus à ARN, Paris, France; 2 CNRS UMR 3569, Paris, France; 3 Université Paris Diderot–Sorbonne Paris Cité, Paris, France; 4 Institut de Biologie et Chimie des Protéines, Molecular Microbiology and Structural Biochemistry, Labex Ecofect, UMR 5086 CNRS, Université de Lyon, Lyon, France; The University of Chicago, UNITED STATES

## Abstract

Over the recent years, several homologues with varying degrees of genetic relatedness to hepatitis C virus (HCV) have been identified in a wide range of mammalian species. HCV infectious life cycle relies on a first critical proteolytic event of its single polyprotein, which is carried out by nonstructural protein 2 (NS2) and allows replicase assembly and genome replication. In this study, we characterized and evaluated the conservation of the proteolytic mode of action and regulatory mechanisms of NS2 across HCV and animal hepaciviruses. We first demonstrated that NS2 from equine, bat, rodent, New and Old World primate hepaciviruses also are cysteine proteases. Using tagged viral protein precursors and catalytic triad mutants, NS2 of equine NPHV and simian GBV-B, which are the most closely and distantly related viruses to HCV, respectively, were shown to function, like HCV NS2 as dimeric proteases with two composite active sites. Consistent with the reported essential role for NS3 N-terminal domain (NS3_N_) as HCV NS2 protease cofactor via NS3_N_ key hydrophobic surface patch, we showed by gain/loss of function mutagenesis studies that some heterologous hepacivirus NS3_N_ may act as cofactors for HCV NS2 provided that HCV-like hydrophobic residues are conserved. Unprecedently, however, we also observed efficient intrinsic proteolytic activity of NS2 protease in the absence of NS3 moiety in the context of C-terminal tag fusions via flexible linkers both in transiently transfected cells for all hepaciviruses studied and in the context of HCV dicistronic full-length genomes. These findings suggest that NS3_N_ acts as a regulatory rather than essential cofactor for hepacivirus NS2 protease. Overall, unique features of NS2 including enzymatic function as dimers with two composite active sites and additional NS3-independent proteolytic activity are conserved across hepaciviruses regardless of their genetic distances, highlighting their functional significance in hepacivirus life cycle.

## Author summary

Despite remarkable progress in the development of therapeutic options, more than 70 million individuals are chronically infected by hepatitis C virus (HCV) worldwide and major challenges in basic and translational research remain. Phylogenetically-related HCV homologues have recently been identified in the wild in several mammalian species, whose host restriction and potential for zoonosis remain largely unknown. We comparatively characterized the functions and properties of nonstructural proteins 2 (NS2) from several animal hepaciviruses and HCV. We demonstrated that NS2 from animal hepaciviruses, like HCV NS2, are cysteine proteases, which function as dimers with two composite active sites to ensure a key proteolytic event of the single viral polyprotein at the NS2/NS3 junction. In addition to the activation of HCV NS2 protease by NS3 N-terminal domain, our data revealed a novel NS3-independent substrate specificity and efficient intrinsic proteolytic activity of NS2. The conservation of its properties and peculiar mode of action among distantly related hepaciviruses supports an important regulatory role for NS2 protein in the life cycle of these viruses. It also strengthens the value of animal, notably rodent hepaciviruses for the development of surrogate, immunocompetent models of HCV infection to address HCV-associated pathogenesis and vaccine strategies.

## Introduction

Approximately 63–79 million individuals were estimated to be chronically infected by hepatitis C virus (HCV) worldwide in 2015 and are at risk of developing severe liver disease including fibrosis, cirrhosis and hepatocellular carcinoma. While the total number of HCV infections is expected to decline or remain flat in many countries thanks to the new era of direct acting antiviral agents (DAAs), HCV-related mortality and morbidity is expected to increase as the infected population ages and progresses to more advanced liver diseases [1]. There is no vaccine available and major challenges in basic, translational and clinical research remain. HCV is a single-stranded positive sense RNA virus belonging to the *Hepacivirus* genus of the *Flaviviridae* family. Until recently, the only known members of the *Hepacivirus* genus were HCV and GB virus B (GBV-B), a hepatotropic virus of unknown origin identified in experimentally infected New World primates [2]. GBV-B infected tamarins (*Saguinus* species) and marmosets (*Callithrix* species) generally develop acute self-resolving hepatitis [3], although several cases of chronic infections have also been reported, highlighting the value of this animal model [4–6]. Over the recent years, a growing number of phylogenetically-related HCV homologues have been identified in the wild in a wide range of mammalian species, including horses [7,8], cattle [9,10], rodents [11–13], bats [14] and Old World primates [15]. Recent studies demonstrated that equine hepaciviruses cause mild hepatic disorders and may establish protracted infections [16–18]. Further epidemiological, pathogenesis and molecular studies are awaited to assess whether these recently identified hepaciviruses have a potential for zoonosis.

**Fig 1:**
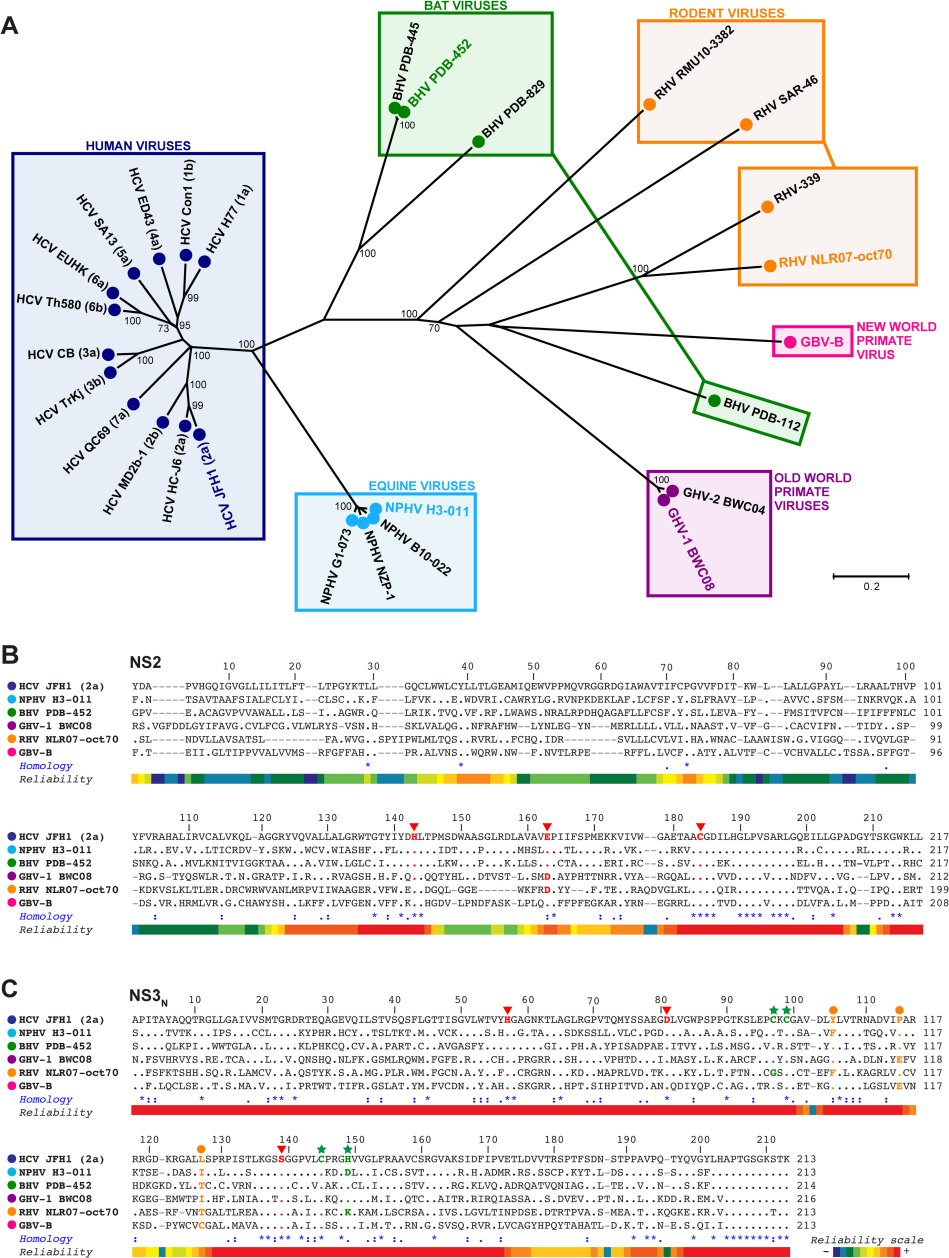
Phylogenetic analysis and multiple sequence alignments of NS2 and NS3 N-terminal domains of selected members of the *Hepacivirus* genus. NS2-NS3_N_ amino acid sequences of selected members of the *Hepacivirus* genus (for details, see Materials and Methods) were aligned with the T-coffee multiple sequence alignment program [74] (A) A phylogenetic tree was constructed using the neighbor joining method under the Jones-Thornton-Taylor model of amino acid substitution implemented in the MEGA6 program [75]. Bootstrap resampling from 2,000 replicates was performed in order to evaluate the reliability of grouping and significant values (>70%) are shown. The tree is drawn to scale with branch lengths proportional to the average number of amino acid substitutions per site, as indicated by the scale bar. Colored boxes cluster viruses according to their experimental (GBV-B) or natural (other viruses) hosts, as indicated above boxes. Viral strains considered in this study are highlighted in the respective species-coded colors. (B-C) The alignments of NS2 (B) and NS3_N_ (C) sequences of the indicated hepacivirus species representatives are shown with respect to the HCV JFH1 (2a) reference sequences with amino acids numbered according to their positions within respective proteins. Gaps between sequences are indicated by hyphens. To highlight the amino acid conservation at each position, residues identical to HCV JFH1 are indicated by dots. In the *Homology* line, identical, highly similar and similar residues across all sequences are symbolized by asterisks, colons and dots, respectively, according to Clustal W conventions. The local robustness of the sequence alignments is displayed in the *Reliability* line, according to a color code from low (blue) to high (red) confidence scores, as illustrated in the reliability scale. Residues comprising the HCV and GBV-B NS2 (B) and NS3 (C) protease catalytic triads and corresponding residues in other hepacivirus sequences are shown in boldface red type as indicated by the red arrowheads. The Cys and His residues of HCV NS3_N_ residues that coordinate Zn^2+^ and aligned residues in the other hepacivirus sequences are shown in boldface green type as indicated by the green stars. The HCV residues forming hydrophobic NS3 surface patch and aligned residues in the other hepacivirus sequences are shown in boldface orange type as indicated by the orange dots.

GBV-B and the newly discovered hepaciviruses share between 25 and 50% nucleotide sequence identity with HCV. The equine nonprimate hepacivirus (NPHV), the best characterized of the novel hepaciviruses, is HCV genetically closest known relative, whereas GBV-B is one of the hepaciviruses most distantly related to HCV (Fig 1 and S1 Fig). The HCV genome consists of a 9.6 kb positive-strand RNA molecule and encodes a polyprotein precursor that is cleaved co- and post-translationally by cellular and viral proteases to yield the capsid protein (C) and the two envelope glycoproteins (E1 and E2), as well as seven nonstructural (NS) proteins (p7, NS2, NS3, NS4A, NS4B, NS5A and NS5B). Several studies demonstrated that, despite limited sequence homology, GBV-B and HCV share a common genomic organization including enzymatic functions [19–22]. Although experimental studies are not as extensive, the recently identified hepaciviruses and particularly the equine NPHV are predicted to have a genomic organization similar to HCV and GBV-B [23], including an internal ribosome entry site (IRES) in their 5' nontranslated regions (5’NTRs) [24–26] and binding sites for miR-122, an essential host factor for viral RNA translation and/or replication [18,27,28]. In addition, the NS3-4A serine proteases of several hepaciviruses have been reported to disrupt host innate immune responses through the cleavage of mitochondrial antiviral signaling protein (MAVS) [29–33]. Mechanisms involved in HCV particle morphogenesis and release, such as capsid protein maturation and lipid droplet targeting [34–36], as well as p7 ion channel activity [37–39] are also shared by GBV-B and NPHV, although GBV-B appears to be uniquely endowed of a more complex and larger (p13) ion channel protein [40].

Through the comparison of several mammalian hepaciviruses, the study undertaken here aimed at identifying conserved or divergent features of the hepaciviral life cycle that could provide new insights into the molecular mechanisms of HCV entry, replication and particle morphogenesis, as well as build bases towards the establishment of immunocompetent surrogate models that would ideally rely on closely-related rodent hepaciviruses. More precisely, the present study focuses on NS2, which in HCV is a 217 amino acid (aa) transmembrane protein that carries a cysteine protease activity responsible for the cleavage of the viral polyprotein at the NS2/NS3 junction [41,42] and is essential for particle morphogenesis through mechanisms that remain incompletely understood [43,44]. NS2 proteolytic activity is a key step in HCV life cycle since NS2/NS3 cleavage is prerequisite for genome replication, NS2 function in particle morphogenesis and NS5A hyperphosphorylation [45–47]. However, two features of NS2 proteolytic mode of action remain intriguing. On the one hand, the crystal structure of HCV NS2 C-terminal catalytic domain revealed an unexpected dimeric protease with two composite functional active sites, highlighting a unique mode of action of this protease [48]. On the other hand, although HCV NS2 C-terminal domain contains the cysteine-based catalytic triad, NS2 was reported to exhibit very low intrinsic protease activity and to require NS3 N-terminal domain for efficient processing at the NS2/NS3 junction [41,42,49].

To gain insight into the properties of hepacivirus NS2 proteases, we carried out a comparative study of the proteolytic modes of action and regulatory mechanisms of NS2 from a selection of genetically divergent hepaciviruses. Following our recent work showing that GBV-B NS2 is a cysteine protease that shares common topological organization with HCV NS2 [22], we report here that NS2 from equine, bat, rodent and Old World primate hepaciviruses are also cysteine autoproteases. By using an approach based on the coexpression of proteolytically inactive NS2-NS3 precursors in mammalian cells, we demonstrated that GBV-B and NPHV NS2 are also dimeric proteases with two composite active sites, highlighting the conservation and the functional significance of this peculiar mode of action among distantly related members of the *Hepacivirus* genus. In addition, we studied the role and virus specificity of NS3 N-terminal domain as NS2 protease cofactor in both transiently transfected and infected cells. Our data revealed unexpected substrate specificity and efficient intrinsic proteolytic activity of HCV NS2 that we further found to be conserved across hepaciviruses.

## Results

### NS2 of newly identified hepaciviruses are cysteine proteases

In order to determine and characterize the function of NS2 from various hepaciviruses, we initially carried out comparative sequence analyses of NS2 and of the N-terminal region of NS3 (NS3_N_) from selected strains of HCV, GBV-B and related viruses recently identified in horses (nonprimate hepaciviruses, NPHV), bats (bat hepaciviruses, BHV), rodents (rodent hepaciviruses, RHV) and Old World primates (Guereza hepaciviruses, GHV). The phylogenetic analyses of these genomic regions illustrate the wide diversity of the *Hepacivirus* genus (Fig 1A) and demonstrate virus clustering that does not necessarily follow species-specific virus segregation. Virus clustering was similar whether NS2-NS3_N_ (Fig 1A), NS3 helicase (S1A Fig), or NS5B RNA-dependent RNA polymerase (S1B Fig) aa sequences were considered. NS3 helicase and NS5B are the most conserved proteins across HCV genotypes and are generally used to address phylogenetic links among HCV strains [50]. Interestingly, the currently known strains of NPHV present a restricted genetic diversity, which is notably lower than the divergence between HCV genotypes. In contrast, wide sequence heterogeneity was observed among BHV and RHV strains, which appears unrelated to host tropism [11,14]. NPHV is HCV closest relative. BHV strains are also phylogenetically close to HCV with the exception of BHV PDB-112 isolate (Fig 1A and S1 Fig). More precisely, NPHV NS2 shares approximately 39% aa identities and 71% aa similarities with HCV NS2, while BHV NS2 shares 31% aa identities and 59% aa similarities with HCV NS2 (S2 Fig). GHV, RHV and GBV-B NS2 are more distantly related to HCV NS2, exhibiting only 20–24% aa identities and 47–52% aa similarities (S2 Fig).

Based on these phylogenetic analyses, we selected one representative hepacivirus strain for each mammalian species to perform a comparative experimental study of NS2 properties: HCV (JFH1), NPHV (H3-011), BHV (PDB-452), GHV (BWC08) and RHV (NLR07-oct70) (Fig 1A). HCV and GBV-B NS2 contain 217 and 208 residues, respectively [22,40,41], and the selected strains of NPHV, BHV, GHV and RHV are predicted to contain 217, 217, 212 and 199 residues, respectively, based on both predicted signal peptidase cleavage sites and sequence homology with HCV and GBV-B. The alignment of NS2 aa sequences from the selected hepaciviruses showed that NS2 N-terminal domains (corresponding to HCV NS2 aa 1–93) are poorly conserved, whereas a greater degree of aa homology is found within the C-terminal domains (Fig 1B and S2 Fig). In particular, His 143 and Cys 184 residues of HCV NS2 protease catalytic triad are fully conserved within hepaciviral sequences and the third negatively charged catalytic residue (HCV Glu 163) is either identical (NPHV, BHV and GBV-B) or highly similar (Asp for GHV and RHV, Fig 1B). In addition, a proline residue with a *cis*-peptide conformation at position 164 of HCV NS2, which is suspected to have a critical role in NS2 catalytic activity by establishing the correct geometry of the Glu 163 side chain [48], is fully conserved within hepaciviral sequences (Fig 1B). NS3 N-terminal region (NS3_N_, aa 1–213 in HCV) presents a higher degree of homology across hepaciviruses than NS2 does, including the conservation of His, Asp and Ser residues forming the catalytic triad of NS3 serine protease in HCV (Fig 1C and S2 Fig). NS3_N_ and in particular a Zn^2+^ binding site within HCV NS3 protease domain that stabilizes NS3_N_ structure was previously demonstrated to be critical for an efficient processing at the NS2/NS3 junction [49]. The HCV NS3 residues that coordinate Zn^2+^ atom (Cys 97, Cys 99, Cys 145 and His 149) are either conserved among other hepacivirus sequences or possibly substituted by cysteine residues in the immediate vicinity for NPHV and RHV (Fig 1C). Altogether, these observations prompted us to determine whether despite limited sequence homology, NS2 of the recently described hepaciviruses displayed proteolytic activity, as previously demonstrated for HCV and GBV-B NS2 [22,41,42].

**Fig 2:**
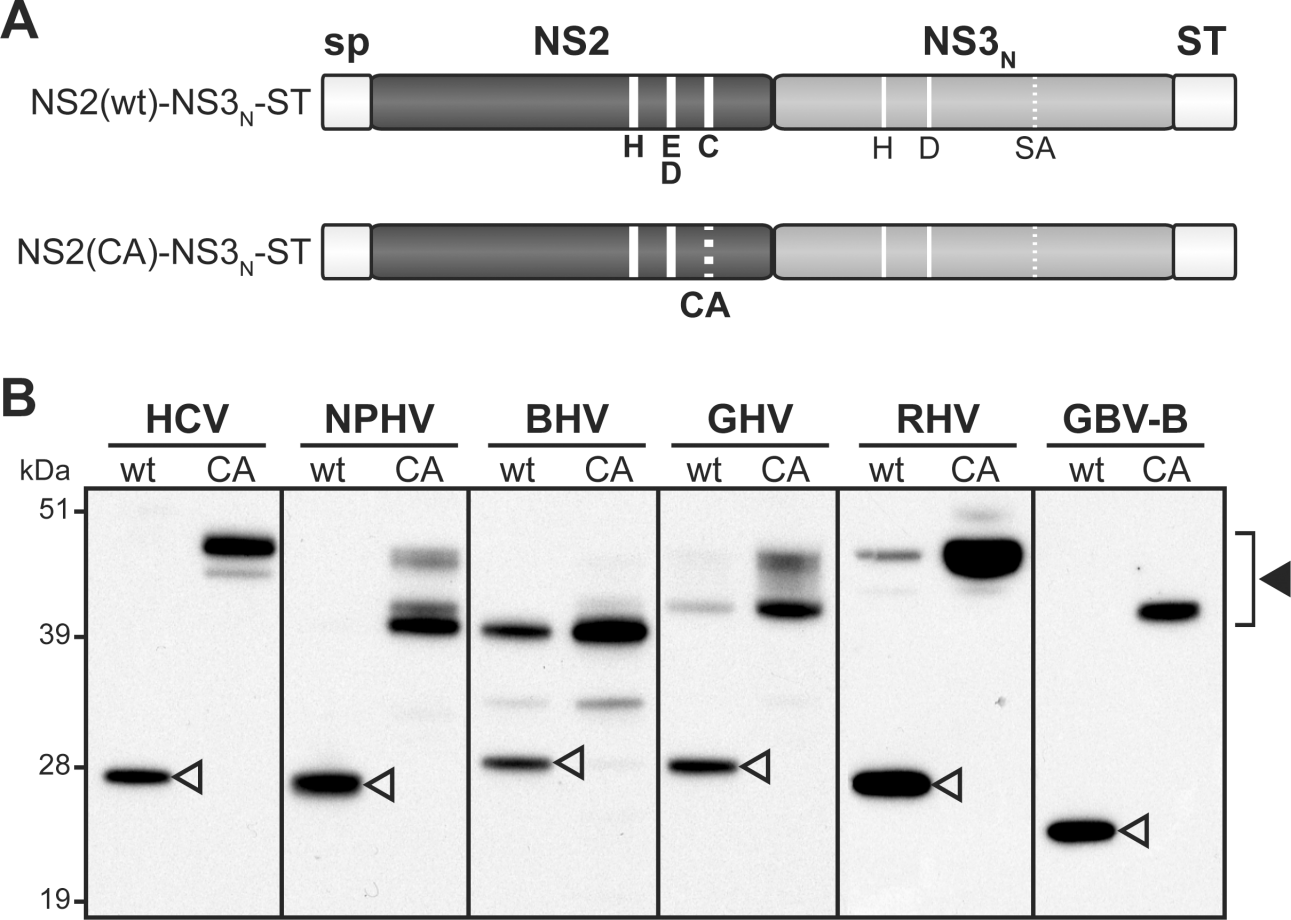
Hepacivirus NS2 autoprotease activity. (A) Schematic representation of the hepacivirus NS2-NS3_N_-ST precursors. Precursors spanning NS2 and NS3 N-terminal domains (NS3_N_) were expressed downstream of a heterologous signal peptide (sp) and C-terminally fused to Strep-tag (ST). NS3_N_ comprises two amino acid residues (H, D; thin solid lines) of putative or established NS3 protease catalytic triads, whereas the third residue (Ser) was mutated into Ala (SA, thin dotted line). NS2(wt)-NS3_N_–ST precursors contain the native residues of the putative or established NS2 protease catalytic triads (H, E/D and C, thick solid lines). NS2(CA)-NS3_N_-ST precursors bear an Ala substitution of the catalytic Cys residue (CA, thick dotted line) in NS2. (B) Proteins extracted from cells transfected with pCMV/NS2(wt)-NS3_N_-ST or pCMV/NS2(CA)-NS3_N_-ST DNAs, allowing expression of native or mutated NS2-NS3_N_-ST precursors of the indicated viruses, respectively, were separated by SDS-PAGE and probed with anti-ST antibodies. Uncleaved precursors and cleaved products are indicated by closed and open arrowheads, respectively.

Toward this aim, we designed a series of plasmid DNAs that allowed the transient expression of NS2-NS3_N_ precursors from the 6 selected hepaciviruses. These polypeptide precursors were expressed downstream of the CD5 signal peptide in order to mimic ER translocation of NS2 N-terminus and their C-termini were fused to a twin Strep-tag (ST) for detection purposes (Fig 2A). Proteins extracted from DNA-transfected 293T cells were separated by SDS-PAGE and ST-reactive products were detected by immunoblotting. For all recently discovered hepaciviruses, like for HCV and GBV-B, wild-type NS2(wt)-NS3_N_-ST precursors were totally or partially cleaved to generate 23- to 28-kDa products, corresponding to NS3_N_-ST (Fig 2B). The contribution of NS3 serine protease to these cleavage events could be ruled out since the conserved, presumed catalytic Ser residue of NS3 was mutated into Ala in all precursors (Fig 2A). Moreover, the proteolytic events observed were abrogated when precursors containing mutated NS2 in which the Cys residue of putative catalytic triads was substituted for Ala (CA) were used. This led to the detection of products with higher molecular masses corresponding to uncleaved NS2-NS3_N_-ST precursors (Fig 2B). For HCV, NPHV, GHV, RHV and GBV-B, the apparent molecular masses of NS2-NS3_N_ uncleaved precursors were as expected according to coding sequences used (S1 Table) and polypeptide calculated molecular masses (S2 Table). In addition, for NPHV, GHV and to a lesser extent for HCV, polypeptides with lower molecular masses were also detected. For BHV, the full-length NS2-NS3_N_ uncleaved precursor was not detected, while a truncated polypeptide of approximately 40 kDa was identified (Fig 2B). It is unlikely that these truncated products resulted from aberrant internal translation initiation since codon-optimized genes were used. These products may rather result from cleavage events by unidentified cellular proteases acting at cryptic sites located within NS2 N-terminal domains, as previously reported for the JFH1 strain of HCV [22,44]. It is, however, important to note that these additional cleavage events did not interfere with the analysis of NS2 proteolytic activity. The electrophoretic profiles of all NS3_N_-ST cleaved products were in agreement with calculated molecular masses (S2 Table). Altogether, these data demonstrated that NPHV, BHV, GHV and RHV NS2 proteins, like HCV and GBV-B, exhibit cysteine protease activities responsible for the cleavage at the NS2/NS3 junction.

### N-terminal domain requirement for NS2 proteolytic activity

HCV NS2 contains 3 transmembrane segments located within its N-terminal region and a C-terminal globular cytosolic domain [22,51]. Previous studies demonstrated that for HCV subtypes 1a and 1b, NS2 proteolytic activity is carried by its C-terminal subdomain, whereas the N-terminal transmembrane region is dispensable for NS2/NS3 junction processing [41,42,52]. Since HCV NS2 topological organization was demonstrated or predicted to be shared by GBV-B and recently discovered hepaciviruses, respectively, notably with respect to three N-terminal transmembrane segments [22], we investigated whether NS2 N-terminal domain from HCV-related viruses was required for an efficient catalytic activity.

**Fig 3:**
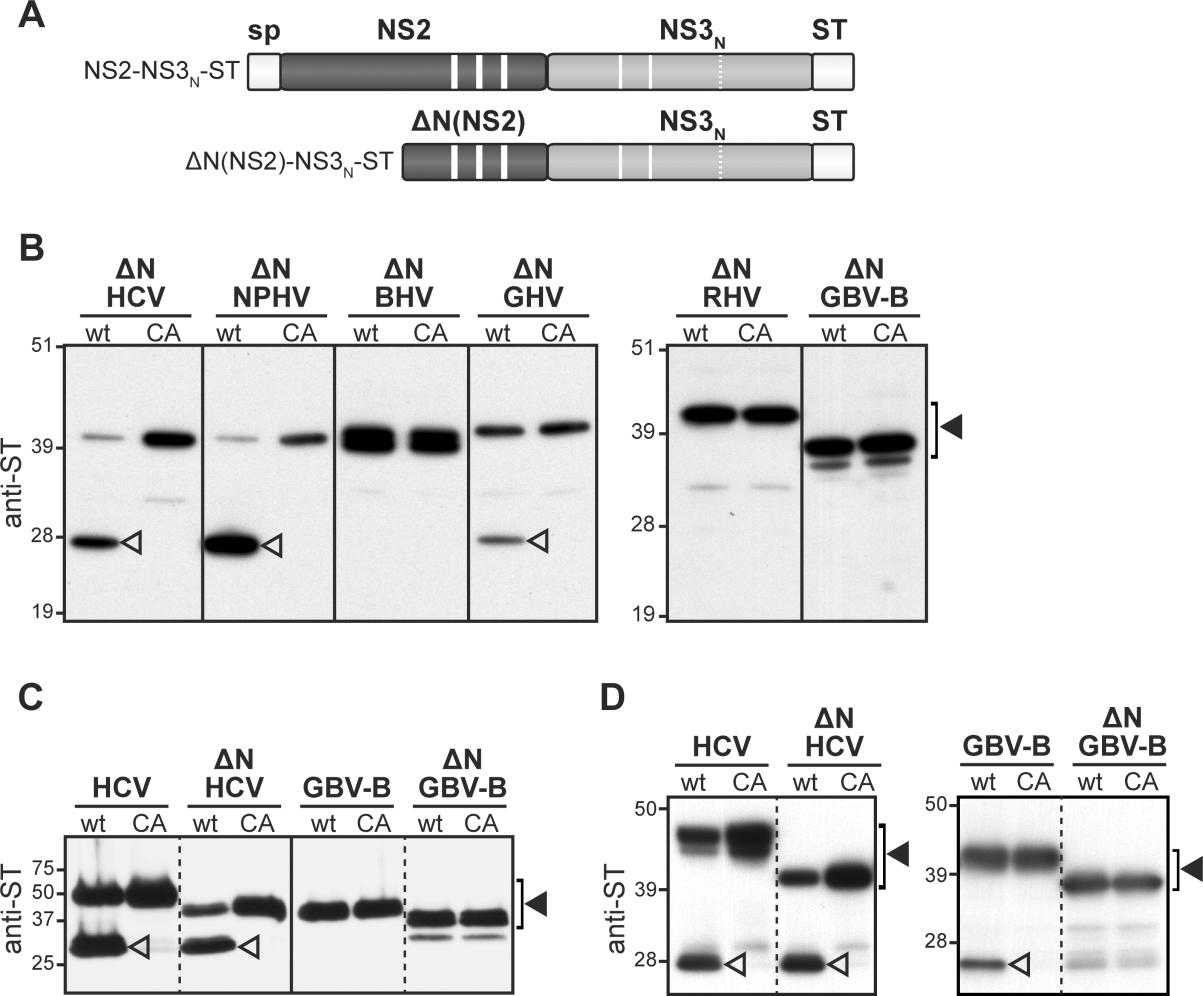
NS2 N-terminal domain requirement for proteolytic activity. (A) Schematic representation of the hepacivirus ΔN(NS2)-NS3_N_-ST truncated precursors, which are derived from NS2-NS3_N_-ST polypeptides (see Fig 1 legend) and lack the hydrophobic N-terminal region of NS2. (B) Proteins extracted from cells transfected with pCMV/ΔN(NS2)(wt)-NS3_N_-ST or pCMV/ΔN(NS2)(CA)-NS3_N_-ST DNAs, allowing the transient expression of native (wt) or mutated (CA) ΔN(NS2)-NS3_N_-ST truncated precursors (ΔN) of the indicated viruses, respectively, were separated by SDS-PAGE and immunodetected with anti-ST antibodies. Uncleaved precursors and cleaved products are indicated by closed and open arrowheads, respectively. (C-D) HCV and GBV-B full-length (HCV, GBV-B) or truncated (ΔN HCV, ΔN GBV-B) precursors were expressed in a wheat germ cell-free expression system in the absence (C) or in the presence (D) of detergent MNG-3. Cell-free expression samples were affinity purified using Strep-Tactin beads, separated by SDS-PAGE and probed with anti-ST antibodies. Uncleaved precursors and cleaved products are indicated by closed and open arrowheads, respectively.

Hepaciviral truncated precursors in which NS2 N-terminal region was deleted [ΔN(NS2)-NS3_N_-ST] were generated (Fig 3A). Immunoblotting of transfected cell extracts using ST-specific antibodies led to the detection of the 6 hepacivirus ΔN(NS2)-NS3_N_-ST truncated precursors with apparent molecular masses of approximately 37 to 41 kDa, as expected (Fig 3B and S2 Table). HCV JFH1 truncated precursor was efficiently cleaved (Fig 3B), extending previous observations to HCV subtype 2a. Similarly, NPHV truncated precursor was cleaved at the NS2/NS3 junction (Fig 3B) with comparable efficiency as full-length precursor (Fig 2B), indicating that NS2 N-terminal region is also fully dispensable for NPHV NS2 catalytic activity. GHV truncated precursor was cleaved, yet with decreased efficiency compared to the full-length precursor (compare Figs 3B and 2B). In contrast, for BHV, RHV and GBV-B, the deletion of NS2 N-terminal region completely abrogated NS2/NS3 cleavage, indicating that NS2 N-terminal hydrophobic domain is required for the catalytic activity of these latter three viral NS2 (Fig 3B).

To address whether proper folding of the membrane-associated N-terminal segments may directly impact NS2 catalytic activity in some hepaciviruses, the proteolytic activity of NS2 from the most distantly related virus, GBV-B, was next compared to that of HCV NS2 in a cell-free expression system using wheat germ extracts [53]. Consistent with the absence of membrane-associated segment requirement (Fig 3B), both full-length and truncated HCV precursors were autoprocessed in this acellular system as in cells, although with overall decreased efficiency (compare Fig 3C with Figs 2B and 3B). While GBV-B truncated precursor was expectedly not cleaved (Fig 3B and 3C), the full-length GBV-B precursor remained totally unprocessed in the cell-free system (Fig 3C), in contrast to complete cleavage in transfected cells (Fig 2B). We recently reported that the addition of 0.1% lauryl maltose neopentyl glycol (MNG-3) detergent to wheat germ extracts greatly improved NS2 solubility and allowed the purification of functional HCV NS2 [53], indicating that this detergent mimicks a membranous environment and supports proper folding of this transmembrane protein. By using wheat germ extracts supplemented with MNG-3, we observed a cleavage of GBV-B full-length precursor, but not of GBV-B truncated precursor (Fig 3D), demonstrating that GBV-B NS2 hydrophobic N-terminal region is required for the proper proteolytic activity of the C-terminal domain of NS2, likely by contributing to critical authentic folding of the protein in a membranous environment. These data highlight structural differences for NS2 protease requirements among hepaciviruses.

### Dimerization of NS2 proteases

**Fig 4:**
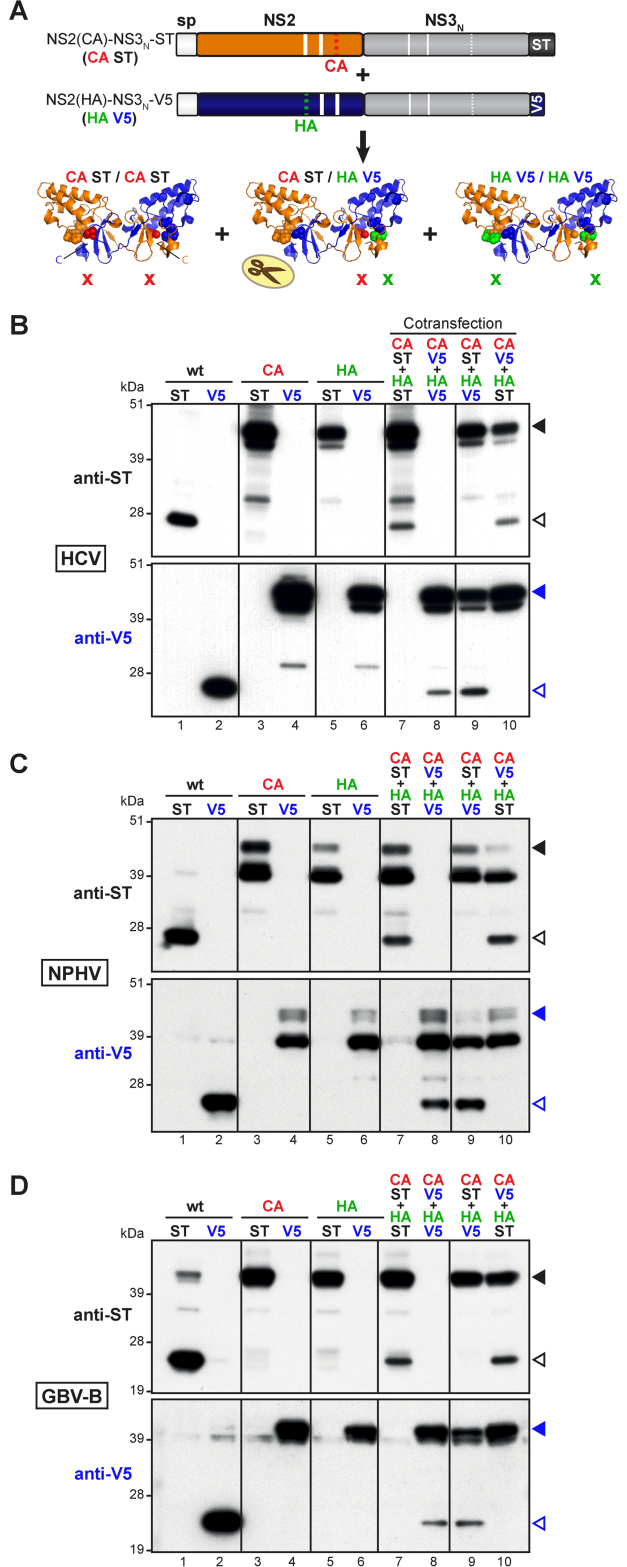
Dimerization of HCV, NPHV and GBV-B NS2 proteases. (A) Schematic representation of NS2 protease dimer formation upon co-expression of mutated precursors. The co-expression of NS2-NS3_N_ precursors (CA ST and HA V5 shown as examples) bearing an Ala substitution of either Cys (CA, red code) or His (HA, green code) catalytic residue and C-terminally fused to Strep-tag (ST, black code) or V5 tag (V5, blue code), respectively, may lead to the formation of NS2-NS3_N_ dimers. Homo- and hetero-dimerizations of NS2 protease, represented according to the three-dimensional crystallographic structure of NS2 catalytic domain (PDB accession number 2HD0) [48], result in the formation of native (wt), single-mutant (HA or CA) or double-mutant (HA and CA) NS2 active sites, as symbolized by scissors, one or two cross signs, respectively. (B-D) Cells were transfected or co-transfected with one (lanes 1 to 6) or two (lanes 7 to 10) pCMV DNAs, allowing the transient expression of NS2-NS3_N_ precursors from HCV-JFH1 (B), NPHV (C) or GBV-B (D). Uncleaved precursors and cleaved products were immunodetected with anti-ST or anti-V5 antibodies (upper and lower images, respectively in each panel) following SDS-PAGE separation of transfected cell extracts and are indicated by closed and open arrowheads, respectively.

The crystal structure of NS2 protease domain from the H77 strain of HCV genotype 1a revealed NS2 dimerization with two composite active sites in which the catalytic His143 and Glu163 residues are contributed by one monomer and the third catalytic partner (Cys184) by the other monomer [48]. These findings were surprising in the context of the early, presumably *cis*-acting cleavage event that NS2 has to carry out during the course of HCV life cycle. We therefore investigated whether NS2 protease dimerization was a unique feature of HCV NS2 or whether this peculiar mode of action was conserved among distantly related hepaciviruses. For this study, we selected three hepaciviruses, the JFH1 strain of HCV genotype 2a, as well as closely and distantly related hepaciviruses NPHV and GBV-B, respectively. Experiments were based on the coexpression of two mutated NS2-NS3_N_ precursors containing an Ala substitution of either the Cys (CA) or the His (HA) residue of NS2 catalytic triads and C-terminally fused to a tag (ST or V5 epitope) (Fig 4A).

As expected, when expressed alone in cells, all ST- and V5-tagged wt precursors were efficiently cleaved at the NS2/NS3 junction (Fig 4B–4D, lanes 1–2), whereas NS2 proteolytic activity was abolished by the introduction of either CA or HA substitution (Fig 4B–4D, lanes 3–6). These results confirm the predicted catalytic role of NPHV NS2 residues His 143 and Cys 184, as well as GBV-B NS2 residues His 138 and Cys 177. Remarkably, the coexpression of CA- and HA-mutated precursors, both tagged with either ST or V5 resulted in partial NS2/NS3 cleavage (Fig 4B–4D, lanes 7–8). These results could only be explained by NS2 protease dimerization and the formation of two composite catalytic triads, with the His and Cys residues of a same active site being contributed by two different monomers. Using the crystallographic 3D structural NS2pro dimeric model described by Lorenz et al. [48], the coexpression of CA- and HA-mutated precursors is indeed expected to lead to the formation of two catalytically inactive CA/CA and HA/HA homodimers, as well as a CA/HA heterodimer (Fig 4A). Based on the putative composite nature of active sites, the CA/HA heterodimer is expected to form one doubly-mutated active site (CA/HA) and one native catalytic site capable of proteolytic activity (Fig 4A, middle structure).

To corroborate our data, we determined which of the CA- and/or HA-mutated NS2-NS3_N_ precursor(s) was(were) cleaved by using one mutated precursor in C-terminal fusion with ST and the other in fusion with V5. Interestingly, only HA-mutated precursors were partially cleaved, whereas CA-mutated precursors remained unprocessed, as shown by immunoblotting using ST- or V5-specific antibodies (Fig 4B–4D, lanes 9–10). These results indicate that for the three hepaciviruses studied, both NS2 C-terminal Leu residue and catalytic Cys residue originate from the same monomer, whereas catalytic His residue is contributed by the second monomer (Fig 4A). Altogether, these data demonstrate that NS2 from the JFH1 strain of HCV genotype 2a, NPHV and GBV-B are dimeric proteases with composite active sites, fully supporting the model previously established for the H77 strain of HCV genotype 1a [48]. The conservation of NS2 dimeric mode of action between distantly related hepaciviruses, although unexpected for a *cis*-acting protease, underscores its functional relevance in the infectious hepaciviral cycle. Consistently, the homology models generated using the crystal structure of HCV 1a NS2 protease [48] revealed overall similar three-dimensional dimeric folds for NS2 protease domains of the various hepaciviruses, including the catalytic pockets (S3 Fig). However, local variations, notably in the length of alpha helices that were shown to associate with membranes in HCV [54], and in the length and orientation of the connecting loops were predicted, with RHV NS2 protease appearing to harbor the widest structural local divergence with respect to HCV and NPHV NS2 proteases. It is worth mentioning that the presence of N-terminal NS2 domains may affect the folding of NS2 C-terminal protease domains and be necessary to regulate or to allow their proteolytic activity in the diverse hepaciviruses (Figs 2 and 3).

### Role and specificity of HCV NS3 N-terminal domain as NS2 protease cofactor

**Fig 5:**
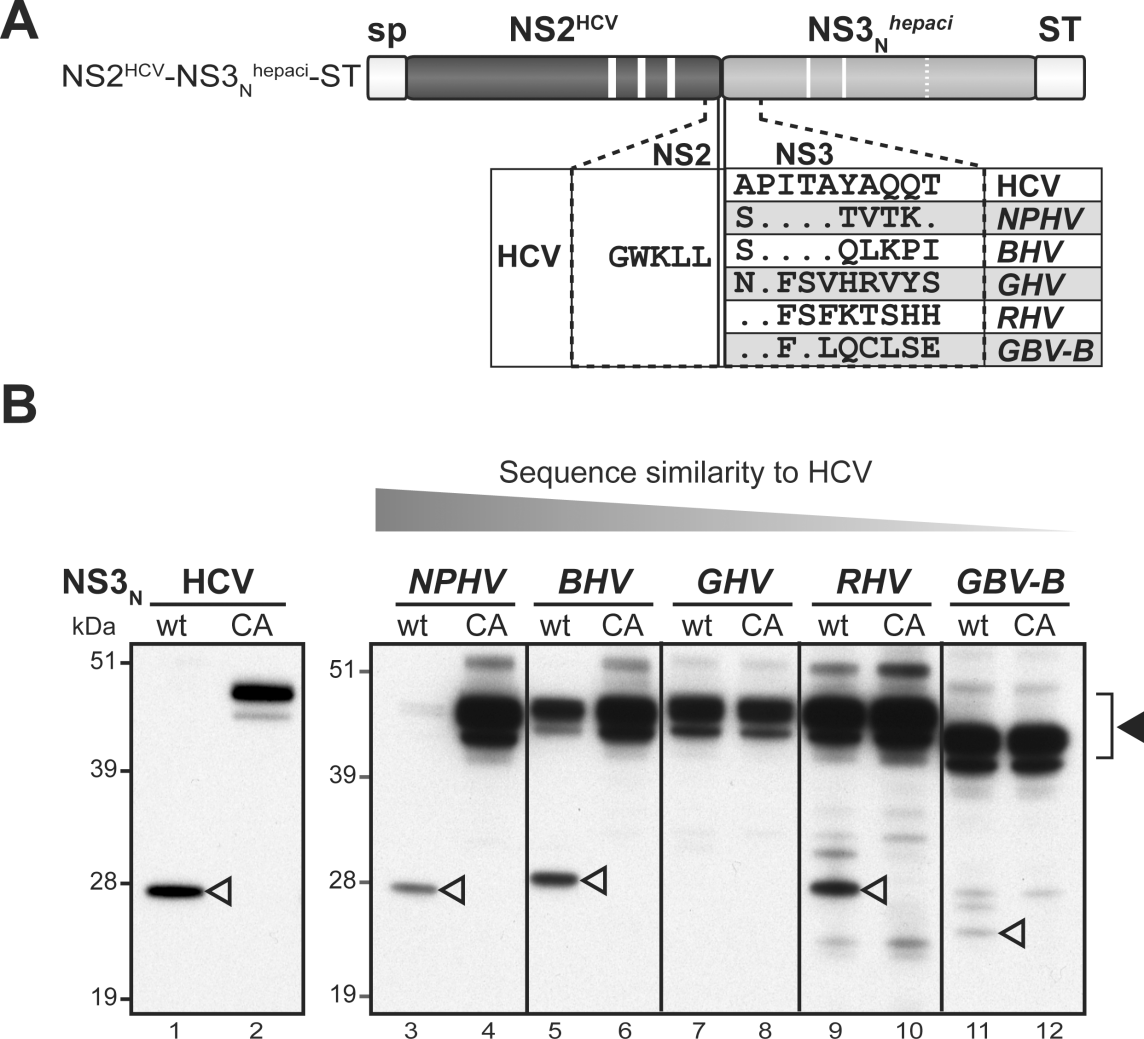
Role and virus specificity of HCV NS3_N_ domain as NS2 protease cofactor. (A) Schematic representation of the chimeric NS2^HCV^-NS3_N_^hepaci^-ST precursors. Precursors spanning NS2 from HCV-JFH1 and NS3_N_ from NPHV, BHV, GHV, RHV or GBV-B (^hepaci^) were expressed downstream of a heterologous signal peptide (sp) and C-terminally fused to Strep-tag (ST). The sequence alignment of NS3 N-terminal sequences from the various hepaciviruses is depicted in the blown-up scheme with respect to HCV corresponding sequence, where identical residues are indicated by dots. (B) Extracts from cells transfected with pCMV/NS2^HCV^(wt)-NS3_N_^hepaci^-ST or pCMV/NS2^HCV^(CA)-NS3_N_^hepaci^-ST DNAs encoding NS2 protease with either native (wt) or mutated (CA) catalytic triad, respectively, were probed with anti-ST antibodies. Uncleaved precursors and cleaved products are indicated by closed and open arrowheads, respectively. The decreasing overall sequence similarity of hepacivirus NS3_N_ with respect to HCV NS3_N_ is represented by a grey triangle.

In order to further characterize the role of NS3_N_ as HCV NS2 protease cofactor, we investigated whether NS3_N_ from divergent hepaciviruses could functionally substitute for HCV NS3_N_ or whether the stimulation of NS2/NS3 cleavage by NS3_N_ was virus-specific. We thus generated chimeric precursors comprising HCV JFH1 NS2 fused to NS3_N_ from NPHV, BHV, GHV, RHV or GBV-B and C-terminally tagged with ST (Fig 5A). As shown in Fig 5B, the chimeric precursor containing NS3_N_ from NPHV was fully cleaved (lanes 3–4), like parental HCV precursor (lanes 1–2). We detected a significant albeit partial cleavage of precursors containing heterologous NS3_N_ from BHV and RHV (lanes 5–6 and 9–10). These data suggested that NS3_N_ from NPHV, BHV and RHV appeared able to stimulate HCV NS2 protease with varying efficiencies. In contrast, NS3_N_ from GHV and GBV-B were not able to significantly activate cleavage at the heterologous NS2/NS3 junction (lanes 7–8 and 11–12). Our data thus indicated that the activation of HCV NS2-mediated cleavage at the NS2/NS3 junction by heterologous hepacivirus NS3_N_ could not be simply explained by overall genetic proximity with HCV, but might depend on the nature of specific residues within heterologous NS3_N_ sequences.

**Fig 6:**
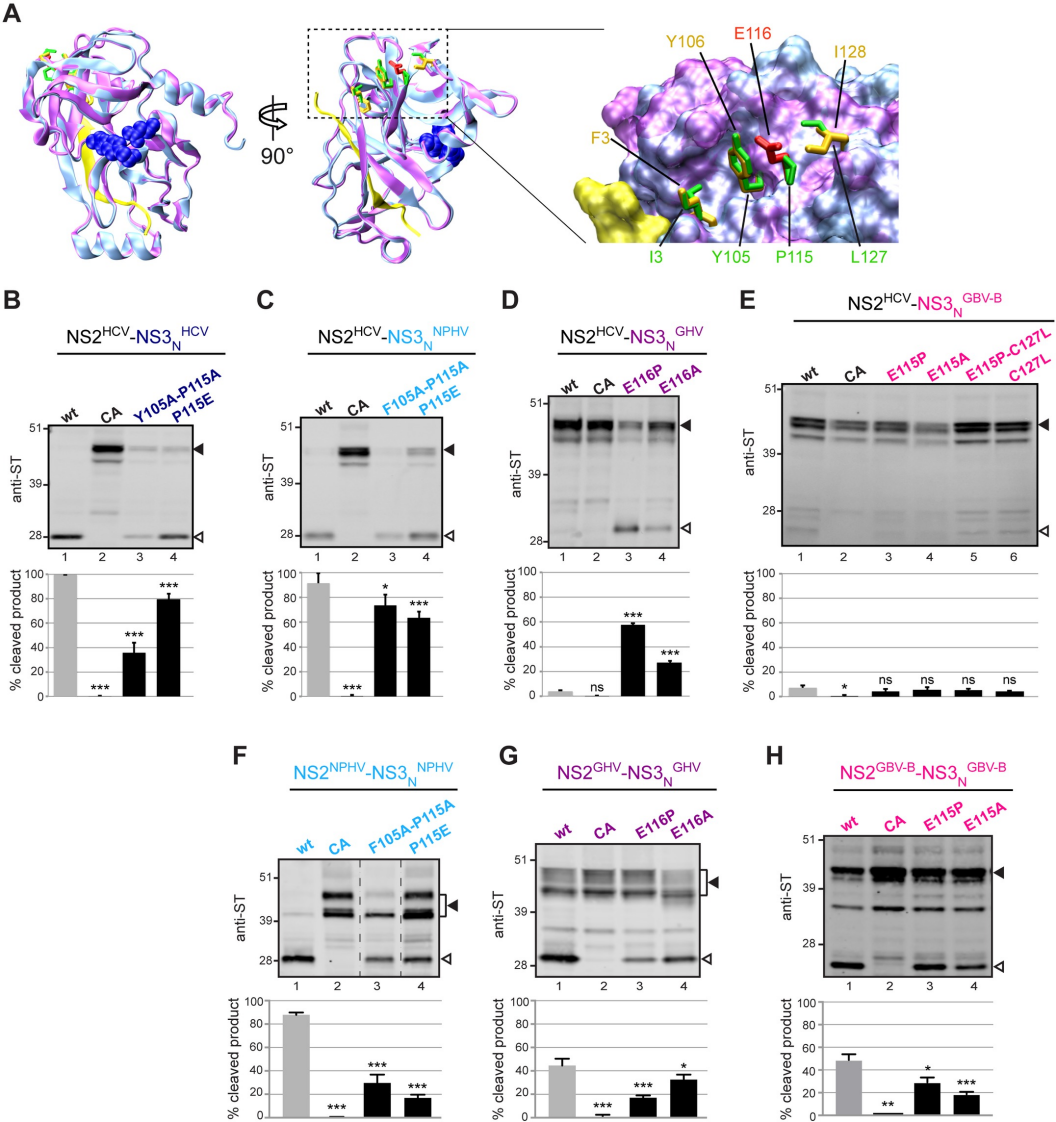
Importance of hydrophobic surface residues in NS3_N_ for HCV NS2 protease activation by heterologous hepacivirus NS3_N_. (A) Superposition of the backbone ribbon structures of GHV NS3_N_ (colored magenta) with HCV JFH1 NS3_N_ (colored cyan) homology models, established by using the crystal structures of HCV NS3 of genotype 1b as template (for details, see Materials and Methods). In the two images at the left (rotated by 90° one with respect to the other), the side-chain atoms of NS3 protease catalytic-triad residues (His 57, Asp 81, and Ser 139/140) are represented as blue spheres of the corresponding van der Waals radii for both models, and NS4A cofactors are represented in yellow. Residues I3, Y105, P115 and L127 in HCV (stick representation, colored green) are shown comparatively to the homologous residues F3, Y106, E116 and I128 (colored orange or red) in GHV. These surface residues are highlighted in the enlargement of NS3_N_ surface patch shown at the right. (B-E) HCV NS2-NS3_N_-ST precursors with either native (wt) or mutated (CA) NS2 catalytic triads and HCV NS2-NS3_N_-ST precursors containing the indicated substitutions at residues 105 and/or 115 of NS3_N_ were expressed in cells (B). Chimeric NS2^HCV^-NS3_N_^hepaci^-ST precursors in which NS2 was derived from HCV and NS3_N_ was derived from NPHV (C), GHV (D), or GBV-B (E) and contained the indicated substitutions at residues 105, 115, and/or 127 were expressed in cells. Chimeric NS2^HCV^-NS3_N_^hepaci^-ST controls harbored native (wt) or mutated (CA) HCV NS2 catalytic triads. (F-G) Hepacivirus NS2-NS3_N_-ST precursors derived from NPHV (F), GHV (G), or GBV-B (H) with either native (wt) or mutated (CA) NS2 catalytic triads and precursors containing the indicated substitutions at residues 105 and/or 115/116 of NS3_N_ were expressed in cells. Transfected cell extracts were probed with anti-ST antibodies. Uncleaved precursors and cleaved products are indicated by closed and open arrowheads, respectively. Dotted lines indicate where lanes originating from the same immunoblot image have been brought together. Quantifications of cleavage rates (% cleaved products over ST-reactive precursors + cleaved products) were performed on 2–5 independent extracts subjected to infrared fluorescent immunoblot imaging and are plotted below representative blot images. Stars above bars represent T test statistical analyses with respect to respective wt controls and are coded as follows: * p<0.01, ** p<0.001, *** p<0.0001, ns: non significant.

Interestingly, a recent study by Isken et al. [47] identified a hydrophobic surface patch in HCV NS3_N_ that promotes HCV NS2 protease stimulation and is potentially critically involved in the coordination of replicase assembly. This hydrophobic patch involves aa residues at positions 3 (Ile), 105 (Tyr), 115 (Pro) and 127 (Leu) of NS3 (Fig 6A). We generated homology models of NS3_N_ from the various hepaciviruses considered in our study using the crystal structure of HCV genotype 1b NS3 protease previously reported [55]. Regardless of the genetic distances, structural models showed overall strikingly similar NS3_N_ folds with relatively minor local structural changes (Fig 6A and S3 Fig). With respect to HCV NS3_N_ hydrophobic surface patch, the examination of hepacivirus NS3_N_ sequence alignment revealed that residues equivalent to HCV Tyr105 are either Tyr or an aromatic residue (Phe) in other hepaciviruses considered in this study (Fig 1C). Interestingly, HCV residue Pro115 is conserved in NPHV, whereas it aligns with the negatively charged residue Glu in GHV and GBV-B (Fig 1C), *i*.*e*. in the two hepacivirus NS3_N_ that were shown to be unable to stimulate HCV NS2 protease (Fig 5B). In NPHV and GHV, the hydrophobic residue Ile at position 127 is similar to HCV Leu127, whereas a cysteine residue is found at the equivalent position in GBV-B and a threonine residue in BHV and RHV (Fig 1C). In order to evaluate the importance of the conservation of NS3_N_ hydrophobic patch for NS3 cofactor activity, we engineered substitutions of residues at positions 105, 115, and/or 127 (or equivalent positions) in NS2(HCV)-NS3_N_(hepaci)-ST precursors and examined autoprocessing of these mutated chimeric precursors using infrared fluorescent revelation of anti-ST immunoblots and quantification of NS2/NS3 junction cleavage efficiency. Dual substitutions of Tyr/Phe105 and Pro115 into Ala residues or single substitutions of Pro115 into Ala or Glu (the latter as found in GHV/GBV-B) introduced in HCV or in chimeric HCV-NPHV precursors resulted in statistically-significant, decreased cleavage efficiency (35–80% cleaved products, as compared to >90% in corresponding parental precursors, Fig 6B and 6C). These data fully support the results obtained by Isken et al. [47], confirming that altering NS3_N_ hydrophobic patch led to a loss in NS3_N_ activating function in HCV. In addition, our results revealed that a similar region in NPHV NS3_N_ is also important for NPHV NS3_N_ cofactor activity. Remarkably, the converse substitution of Glu116 into either Pro, as found in HCV and NPHV, or Ala resulted in a substantial gain of function of GHV NS3_N_ as HCV NS2 stimulating cofactor (55% and 25% cleaved products, respectively, as compared to <5% in corresponding chimeric GHV/HCV precursor, Fig 6D). It should be noted, however, that none of the substitutions designed to introduce HCV-like hydrophobic residues into GBV-B NS3_N_ was sufficient to result in HCV NS2 protease stimulation (Fig 6E). This highlights the greater genetic distance between GBV-B and HCV. Altogether, these data indicate that NS3_N_ of some hepaciviruses are able to activate HCV NS2 protease and that this heterologous stimulation is dependent on NS3_N_ hydrophobic surface patch.

We investigated whether similar surface residues in NS3_N_ of NPHV, GHV and GBV-B were important for the stimulation of cognate NS2 proteases. For this, we engineered substitutions of residues at positions 105 and/or 115 (or equivalent positions) of NS3_N_ in NS2-NS3_N_-ST precursors of NPHV, GHV and GBV-B and examined autoprocessing of these mutated precursors as described above. Cleavage efficiencies were scored by quantification of product/precursor ratios using infrared fluorescent revelation of anti-ST immunoblots (Fig 6F–6H). All mutations resulted in significant loss of NS2 protease activity to varying degrees for the three hepaciviruses. Importantly, these residues lie at a similar surface location in NS3_N_ (Fig 6A and S3 Fig) as the hydrophobic patch previously defined for HCV [47], strengthening the conserved role of this NS3_N_ region for NS2 protease stimulation within the hepacivirus genus.

### Characterization of HCV NS2 protease substrate specificity in the absence of NS3 cofactor domain

**Fig 7:**
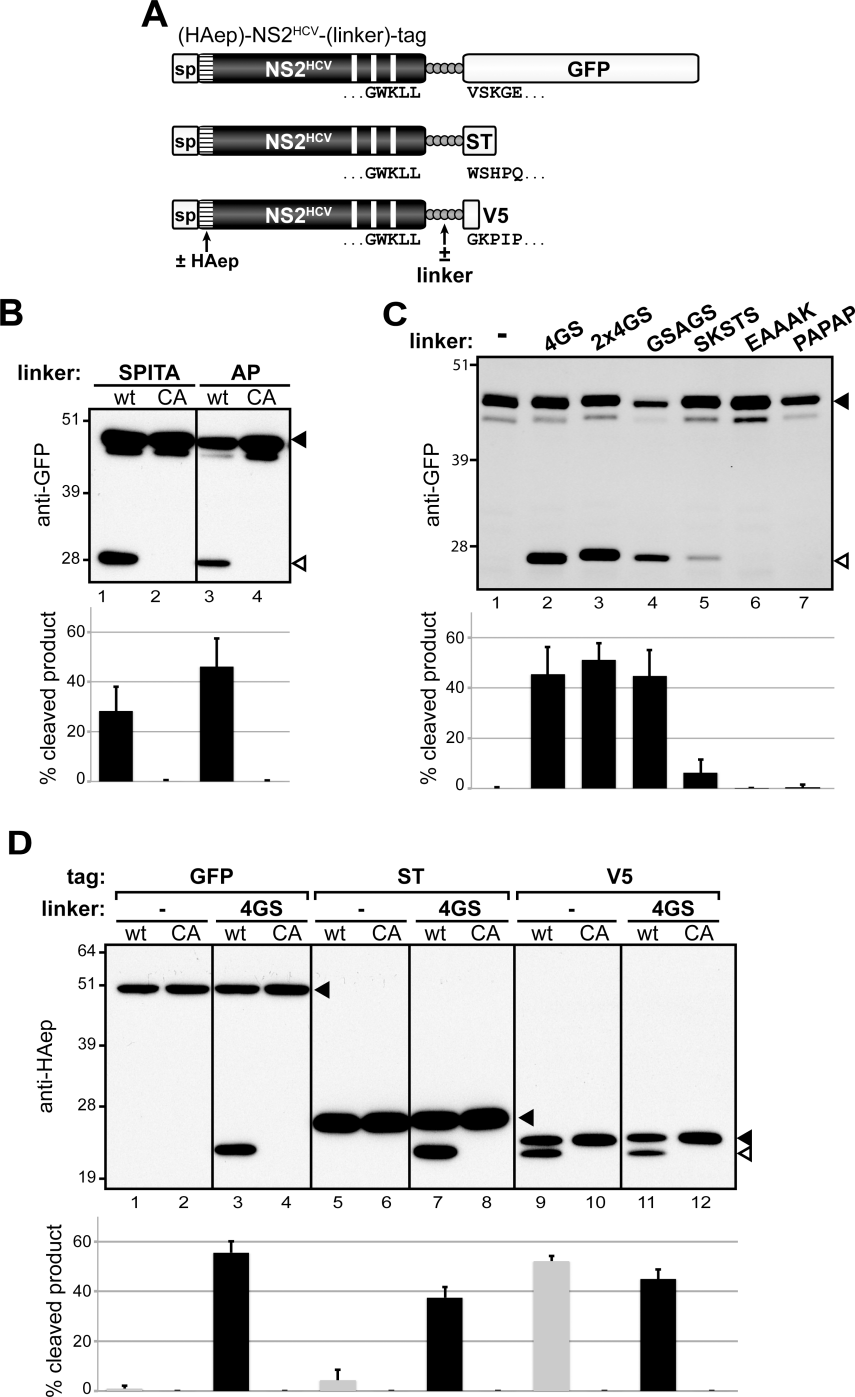
HCV NS2 protease activity in the absence of NS3_N_ cofactor domain. (A) Schematic representation of (HAep)-NS2^HCV^-(linker)-tag precursors. Precursors spanning HCV NS2 C-terminally fused to GFP, ST or V5 (tag), either directly or via a linker, were expressed downstream of a heterologous signal peptide (sp). Precursors used in panel D comprise a HA epitope (HAep, hatched box) introduced downstream of HCV NS2 N-terminal residue. HCV NS2 C-terminal residues, as well as GFP, ST or V5 N-terminal residues are displayed below the corresponding boxes. (B-D) Cells were transfected with pCMV/(HAep)-NS2^HCV^-(linker)-tag DNAs that encode NS2 comprising either native (wt) or mutated (CA) catalytic triad and immediately fused to the linker indicated at the top of the panels or no linker (-) and GFP (B-C) or the indicated tag (D). Transfected cell extracts were probed with anti-GFP (B-C) or anti-HAep (D) antibodies. Uncleaved precursors and cleaved products are indicated by closed and open arrowheads, respectively. Quantifications of cleavage rates (% cleaved products over total HAep-reactive precursors) were performed on 2–3 independent extracts subjected to infrared fluorescent immunoblot imaging and are plotted below representative blot images.

Interestingly, sequence alignment of hepacivirus NS3 N-termini showed that the N-terminal five residues of HCV NS3 are highly conserved in NPHV and BHV sequences, and that the N-terminal two residues are identical in HCV, RHV and GBV-B NS3 (Fig 5A). Since HCV NS2 fused to 2 aa of HCV NS3 was previously reported to support only basal proteolytic activity [49], we undertook to determine to which extent the conservation of NS3_N_ N-terminal residues contributed to cleavage efficiency at heterologous NS2^HCV^/NS3^hepaci^ junctions. We generated precursors containing HCV NS2 followed by NPHV/ BHV NS3 aa residues 1–5 (SPITA) or by RHV/ GBV-B/ HCV NS3 aa residues 1–2 (AP) and C-terminally fused to green fluorescent protein (GFP, 238 aa) (Fig 7A), and we quantified their autoprocessing using anti-GFP antibodies. Surprisingly, these 2 precursors were efficiently cleaved (~30–50%), although not to completion (Fig 7B, lanes 1 and 3). Cleavage was abrogated by inactivation of NS2 catalytic Cys residue (Fig 7B, lanes 2 and 4). This indicated that the fusion of the 5 SPITA or the 2 AP residues downstream of HCV NS2 was sufficient to permit NS2 autoproteolytic activity in the absence of NS3 moiety. Such an efficient cleavage was not anticipated since only an extremely basal proteolytic activity was previously reported for HCV NS2 followed by these few NS3 aa in a different expression system, whereas the N-terminal domain of NS3 was required for a productive processing at the NS2/NS3 junction [49].

Based on the unexpected efficient processing of NS2^HCV^-SPITA-GFP and NS2^HCV^-AP-GFP (Fig 7B), we further examined the nature of residues located immediately downstream of HCV NS2 that may be permissive for NS2 proteolytic activity in the context of GFP C-terminal fusion. A precursor containing HCV NS2 directly fused to GFP remained unprocessed (Fig 7C, lane 1 and Fig 7D, lane 1). In contrast, the fusion of NS2 to GFP via a 5 aa flexible linker comprised of 4 Gly and a Ser (NS2-4GS-GFP) yielded substantial amounts (~50%) of a GFP-reactive polypeptide with a molecular mass close to that of GFP (Fig 7C, lane 2 and Fig 7D, lane 3). This product was not observed upon mutation of NS2 catalytic Cys residue (CA, Fig 7D, lane 4). Such a productive processing was unexpected since the 4GS linker and the downstream GFP heterologous sequences do not present sequence homology with any hepacivirus NS3 N-terminal sequence (Figs 5A and 7A).

In order to determine the sequence requirements for NS2 catalytic activity in the absence of NS3_N_, we next examined the autoproteolytic activity of NS2 precursors fused to GFP via various short linkers selected to introduce flexibility [GGGGSGGGGS (2x4GS), GSAGS and SKSTS] or rigidity (EAAAK and PAPAP) to the polypeptide backbone [56]. The 10-aa (2x4GS) and the 5-aa GSAGS flexible linkers both allowed efficient (~50%) cleavage at NS2 C-terminus (Fig 7C, lanes 3–4). This cleavage was demonstrated to be mediated by NS2 catalytic activity since cleaved products were abrogated upon NS2 CA mutation (S4 Fig). In contrast, the flexible SKSTS or the rigid EAAAK or PAPAP sequences were not appropriate substrates for NS2 catalytic pocket (Fig 7C, lanes 5–7). These data suggested that the composition and the flexibility of the sequence located immediately downstream of NS2, but not necessarily its length were critical for NS2 proteolytic activity in the absence of NS3. In particular, this set of data suggested that the presence of a glycine residue downstream of NS2 C-terminus may promote NS3-independent NS2 catalytic function. Such a glycine residue is not naturally found as the P'1 residue of the NS2/NS3 cleavage site in any reported sequence of hepacivirus. Interestingly, a proline residue is fully conserved as the P'2 residue of the authentic cleavage sites in all hepaciviruses examined. To obtain further insight into the importance of the first 2 residues downstream of NS2 for autoproteolytic cleavage, we engineered HCV NS2-linker-GFP precursors bearing GPGGS, APGGS, NPGGS or SPGGS as linkers. While the former two precursors were cleaved to similar efficiency as NS2-4GS-GFP, cleavage of the latter two precursors was significantly reduced (S5 Fig). These data indicate that a small residue (Gly/Ala) is preferred at the P'1 position, whereas a proline at the P2' position has no effect in a linker-GFP fusion context.

We subsequently analyzed the effect of the sequence located downstream of the 5aa linker on NS2 catalytic activity. For this, we used precursors containing HCV NS2 C-terminally fused to the 4GS linker followed by various tags, GFP (238 aa), Strep tag (ST, 28 aa), or V5 epitope (14 aa) (Fig 7A). In order to be able to compare the cleavage efficiencies of these precursors, a HA epitope (HAep) was introduced at NS2 N-terminus and demonstrated to have no effect on NS2 protease activity (S6 Fig). Immunoblotting using HAep-specific antibodies showed that the 3 precursors were cleaved with similar efficiencies in an NS2 protease-mediated manner, regardless of the nature of the C-terminal tag sequence (Fig 7D, lanes 3–4, 7–8 and 11–12). Of note, the NS2-4GS-GFP precursor demonstrated consistent cleavage efficiencies, whether HAep-NS2 or GFP counterparts of cleaved products were quantified using anti-HAep or anti-GFP antibodies, respectively (Fig 7C, lane 2 and Fig 7D, lane 3). Furthermore, whereas precursors containing NS2 directly fused to GFP or ST remained unprocessed (Fig 7D, lanes 1–2 and 5–6), NS2-V5 precursor was efficiently cleaved (Fig 7D, lanes 9–10). This proteolytic processing in the absence of a linker sequence was probably due to the aa composition of the V5 epitope which N-terminal residue is a glycine (see Fig 7A). Altogether, these results demonstrate for the first time an efficient catalytic activity of HCV NS2 protease in the absence of any NS3 sequence, revealing an unexpected intrinsic proteolytic activity of NS2.

**Fig 8:**
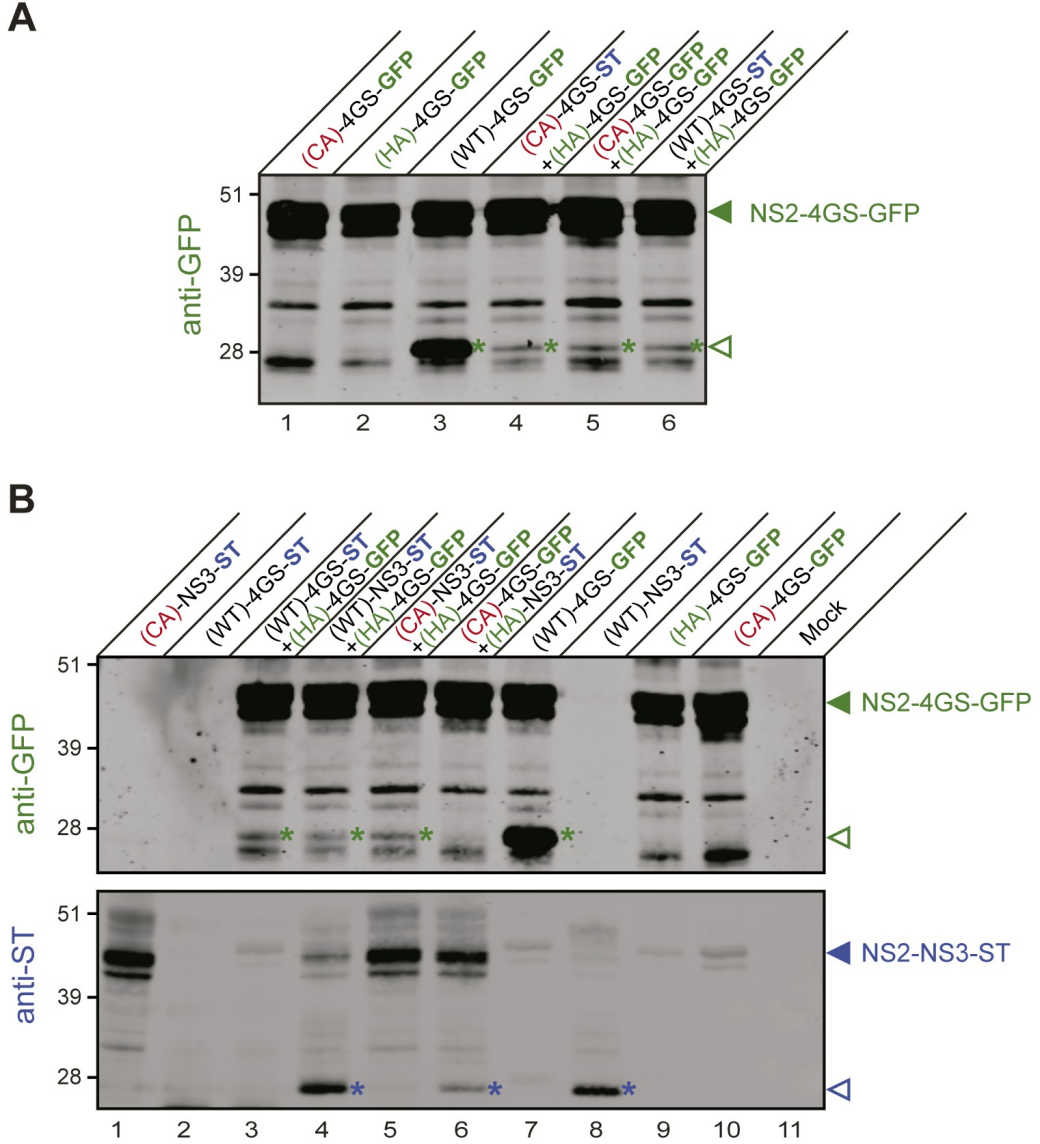
Dimerization and *trans*-cleavage in the linker-tag fusion context. Protein extracts were prepared from cells expressing or co-expressing the indicated fusion polypeptides, which comprise HCV NS2 with either native (WT) or mutated catalytic triads [by alanine substitution of cysteine (CA) or histidine (HA) residues] in C-terminal fusion with the indicated sequences and tag: either HCV NS3 followed by strep-tag (NS3-ST), or linker 4GS followed by GFP (4GS-GFP) or strep-tag (4GS-ST). Protein extracts were separated by SDS-PAGE and probed with anti-GFP antibodies (A; B, upper image) or anti-ST antibodies (B, lower image). Uncleaved precursors and cleaved products are indicated by closed and open arrowheads, respectively. Cleaved products are also marked by color-coded asterisks (GFP-reactive products in green and ST-reactive products in blue).

We next assayed NS2-linker-tag fusion polypeptides in a composite protease assay using GFP and ST as tags, as previously performed with NS2-NS3-ST/V5 substrates (Fig 4). We found that co-expression of catalytically inactive NS2(HA)-4GS-GFP fusion precursor together with catalytically active NS2(WT)-4GS-ST or co-expression of two catalytically inactive linker-tag fusion precursors, e.g. NS2(CA)-4GS-GFP and NS2(HA)-4GS-GFP (same tag) or NS2(CA)-4GS-ST and NS2(HA)-4GS-GFP (different tags) led to partial cleavage on the monomer harboring the catalytic cysteine residue (HA) (Fig 8A, lines 4–6). Interestingly, we also documented partial cleavage of catalytically inactive NS2(HA)-4GS-GFP precursor by native NS2-NS3-ST or by catalytically inactive NS2(CA)-NS3-ST (Fig 8B, lines 4–5). Conversely, partial cleavage of NS2(HA)-NS3-ST was also observed in the presence of catalytically inactive NS2(CA)-4GS-GFP (Fig 8B, line 6). These data thus indicate that NS2 dimerization can also lead to NS2-mediated cleavage in the context of NS2 linker-tag fusion polypeptides.

### The NS3-independent substrate specificity of NS2 protease is conserved among mammalian hepaciviruses

**Fig 9:**
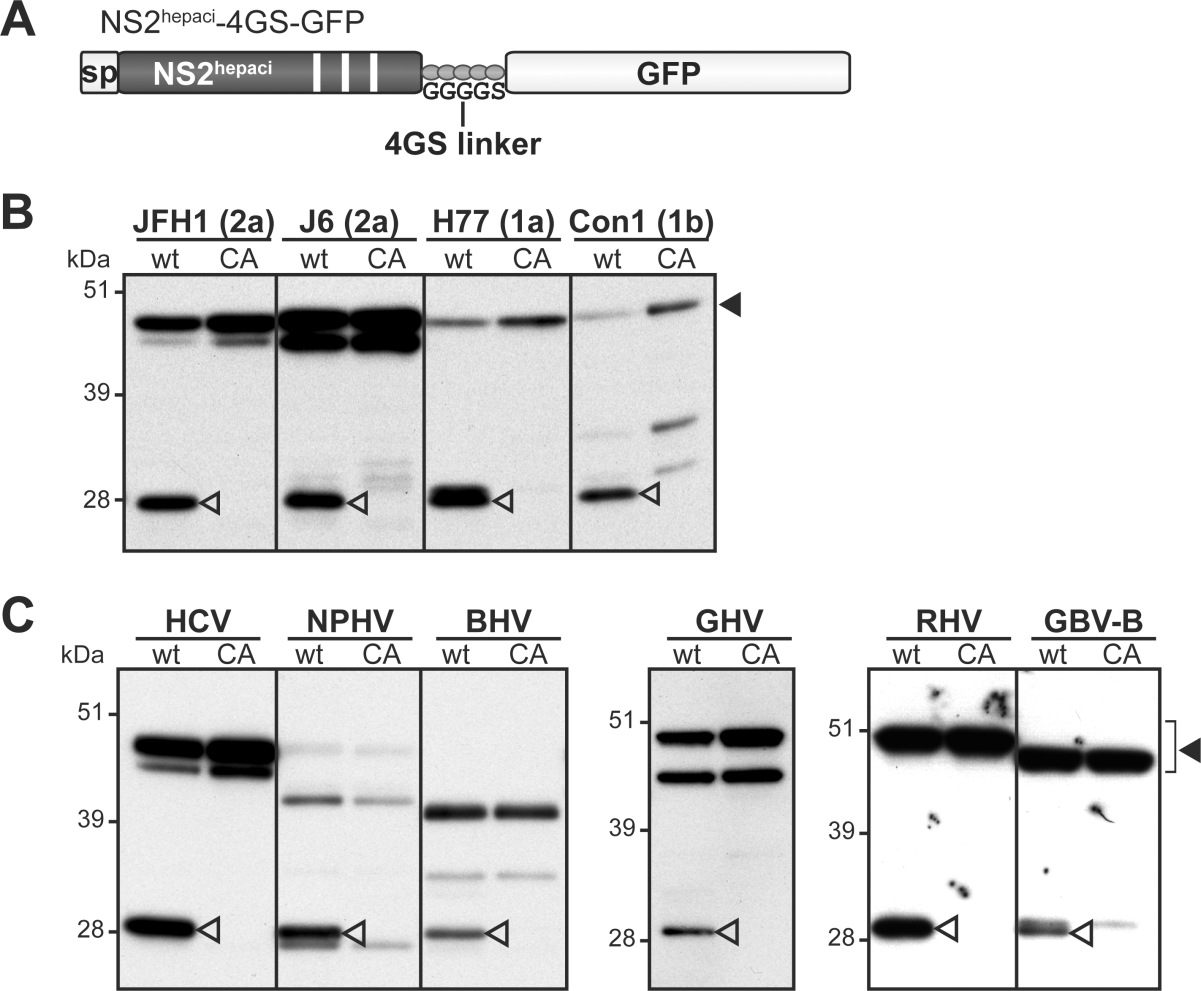
Functionality of NS2 proteases from various hepaciviruses in the absence of NS3_N_ domain. Cells were transfected with pCMV/NS2^hepaci^-4GS-GFP DNAs (A), allowing the transient expression of NS2 from the indicated HCV strains (subtypes in parentheses) (B) or hepaciviruses (C). NS2 proteases comprised either the native catalytic triad (wt) or an Ala substitution of the catalytic Cys residue (CA). Uncleaved precursors and cleaved products were immunodetected with anti-GFP antibodies following SDS-PAGE separation of transfected cell extracts and are indicated by closed and open arrowheads, respectively.

We investigated whether the NS3-independent substrate specificity of NS2 protease was conserved across HCV genotypes as well as more distantly related hepaciviruses. First, we compared the autoproteolytic properties of NS2-4GS-GFP precursors containing NS2 from the JFH1 or the J6 strains of HCV genotype 2a, the H77 strain of HCV genotype 1a or the Con1 strain of HCV genotype 1b (Fig 9A). Anti-GFP immunoblots presented in Fig 9B showed that all wt precursors exhibited substantial to very efficient autoproteolytic activities carried out by NS2 cysteine protease. Similarly, NS2-4GS-GFP precursors containing NS2 from NPHV, BHV, GHV, RHV and GBV-B were all specifically cleaved by NS2 protease in the absence of NS3 (Fig 9C).

Altogether, these data indicate that NS3-independent NS2 protease activity is conserved across HCV genotypes and distantly related hepaciviruses, suggesting that this intrinsic activity is likely to have functional significance in the hepaciviral life cycle and/or hepacivirus/host interactions.

### The NS3-independent proteolytic activity of NS2 is functional in the context of HCV infection

**Fig 10:**
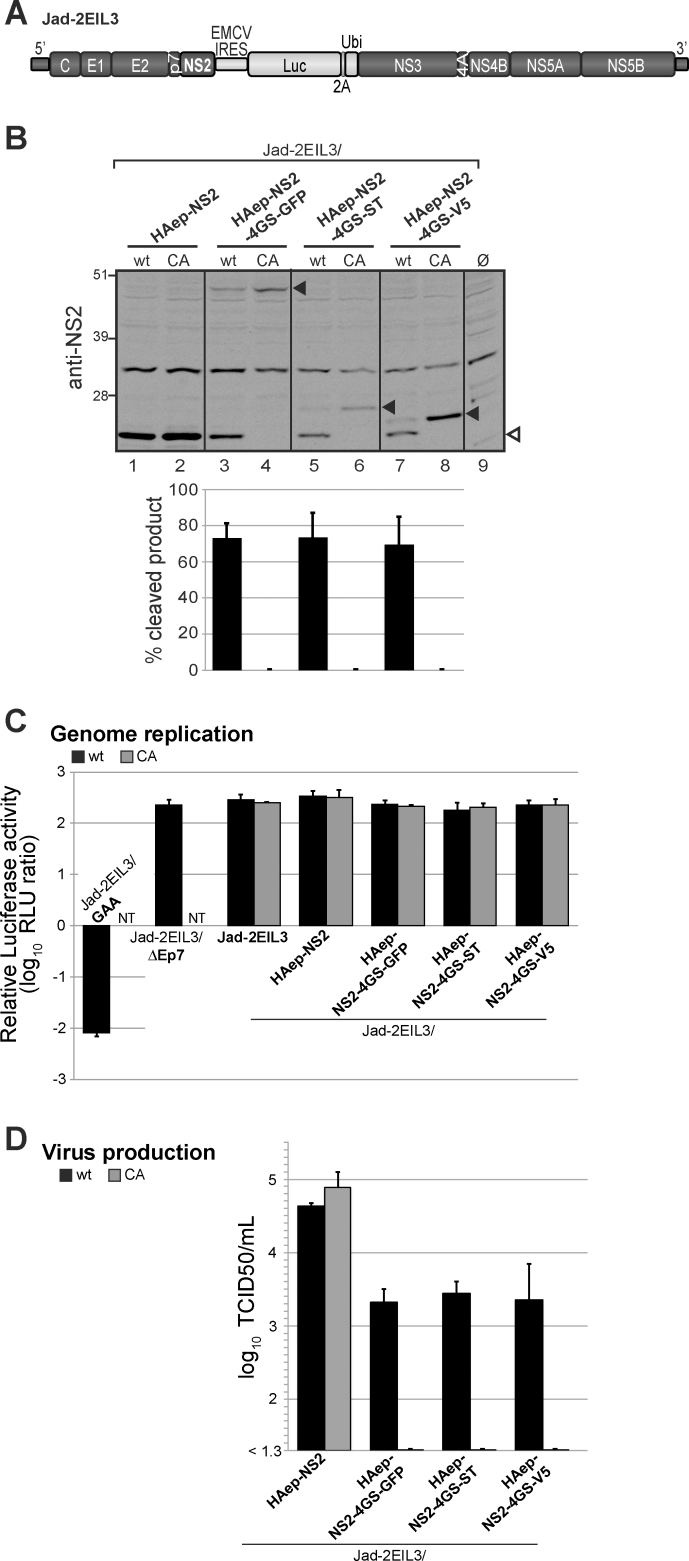
HCV NS2 protease substrate specificity in infected cells. (A) Schematic representation of the parental Jad-2EIL3 cDNA. Genomic sequences derived from a cell culture-adapted JFH1 variant (Jad) are represented by dark grey boxes. The heterologous EMCV IRES sequence followed by the Firefly luciferase (Luc), FMDV 2A peptide (2A) and ubiquitin (Ubi) coding sequences were inserted between NS2 and NS3 coding sequences, and are represented by light grey boxes. (B) Proteins extracted at 72h post-transfection from Huh7.5 cells transfected with Jad-2EIL3/HAep-NS2 or the indicated Jad-2EIL3/HAep-NS2-4GS-tag RNAs encoding NS2 protease with native (wt) or mutated (CA) catalytic triad were analyzed with antibodies specific for JFH1 NS2. Quantifications of cleavage efficiencies following infrared fluorescent imaging are derived from 2 independent experiments. (C) Genome replication. Relative intracellular luciferase activities were determined at 72 h post-transfection and expressed relatively to activities at 4 h post-transfection following transfection of Huh7.5 cells with the indicated RNAs encoding NS2 protease with native (wt, black bars) or mutated (CA, grey bars) catalytic triad. Controls include the parental Jad-2EIL3 RNA, the replication deficient Jad-2EIL3/GAA RNA and the assembly-deficient Jad-2EIL3/ΔEp7 RNA. NT: not tested (D) Infectious viral particle production was quantified by endpoint dilution titration of supernatants collected at 72h post-transfection and is expressed as log TCID50/mL titers. Means ± standard deviations of 3 independent transfections, each in duplicates are shown (C-D).

HCV NS2 is not only an essential protease, but is also required for virion assembly and this function was shown to depend on both N-terminal transmembrane and C-terminal protease domains of NS2, including its C-terminal leucine residue [46]. To further study the relevance of NS3-independent NS2 protease activity in HCV infectious life cycle, we generated a genome-length HCV cDNA derived from a cell culture adapted JFH1 variant [57], in which the heterologous IRES sequence from the murine encephalomyocarditis virus (EMCV) was inserted between NS2 and NS3 coding sequences. This resulted in two translational units, therefore uncoupling NS2/NS3 processing from RNA replication as previously shown [44] and allowing the study of the proteolytic activity of NS2 upon its C-terminal fusion to heterologous sequences. In addition, the Firefly luciferase reporter sequence was introduced downstream of the EMCV IRES followed by the foot and mouth disease virus (FMDV) 2A peptide and the ubiquitin coding sequences in order to facilitate the monitoring of genome replication and infectivity (Jad-2EIL3, Fig 10A). Several cDNAs were engineered in the Jad-2EIL3 backbone and designed to encode NS2 with a HAep at its N-terminus (between NS2 aa 1 and 2) and either C-terminally fused to a 4GS linker followed by GFP, ST or V5 (Jad-2EIL3/HAep-NS2-4GS-tag) or with no C-terminal fusion (Jad-2EIL3/HAep-NS2). Additionally, these cDNAs were created to encode NS2 protease with either a native (wt) or a mutated (CA) catalytic site designed to abrogate NS2-mediated cleavages.

Human hepatoma Huh7.5 cells were transfected with Jad-2EIL3-derived synthetic RNAs. Proteins extracted at 72h post-transfection were analyzed by immunoblotting with NS2-specific antibodies. The three NS2(wt)-4GS-GFP/ST/V5 precursors were specifically cleaved by NS2 protease at comparably high levels regardless of the nature of the C-terminal tag (Fig 10B). Cleavage efficiencies in RNA-transfected cells were of the same magnitude as those observed in the transient expression system (Fig 7D), indicating the relevance of NS2 intrinsic protease activity in both experimental systems.

We further analyzed the effect of these fusions on HCV replication and particle assembly by quantifying luciferase reporter activities at 72h post-transfection in cells transfected with Jad-2EIL3-derived RNAs and at 72h post-infection in cells infected with supernatants collected at 72h post-transfection, respectively. All RNAs replicated efficiently, similarly to Jad-2EIL3 parent and in contrast to a replication-deficient Jad-2EIL3/GAA RNA encoding inactivating mutations in the polymerase active site (Fig 10C). This was in agreement with the fact that the EMCV IRES insertion between NS2 and NS3 coding sequences rendered bicistronic RNA replication independent of NS2 proteolytic activity [44]. In contrast to an assembly-deficient RNA devoid of E1-E2-p7 coding sequences (Jad-2EIL3/ΔEp7), Jad-2EIL3 and Jad-2EIL3/HAep-NS2 RNAs encoding wt or CA-mutated NS2 proved equally infectious (S7 Fig). This indicated that both the insertion of a HAep downstream of NS2 N-terminal residue and the Ala substitution of NS2 catalytic Cys residue did not alter NS2 function in particle morphogenesis, extending previous observations [43,44,51]. Remarkably, whereas the transfection of RNAs encoding NS2(wt)-4GS-tag polypeptides led to high-level infectious virus production, the transfection of corresponding RNAs encoding CA-mutated NS2 did not yield TCID50-measurable infectious production (Fig 10D). Thus, the lack of infectious particle production from RNAs encoding inactive NS2 could conclusively be attributed to the C-terminal fusion of NS2 to 4GS-tags, which impaired NS2 function in particle assembly. Conversely, wt NS2 autoproteolytic activity leading to the release of functional, C-terminally untagged HAep-NS2 was corroborated by efficient infectious virus production. Accordingly, the ~ 1 log unit decrease in infectivity observed with Jad-2EIL3/HAep-NS2(wt)-4GS-tag RNAs compared to Jad-2EIL3/HAep-NS2(wt) (Fig 10D) may be explained by the lower intracellular levels of assembly-competent HAep-NS2, as HAep-NS2(wt)-4GS-tag polypeptides were not cleaved to completion (Fig 10B). In conclusion, these results concur to demonstrate that HCV NS2 substrate specificity initially unveiled in a transient expression system also operates in infected hepatoma cells, and that NS3_N_ is not a mandatory cofactor for NS2 protease activity.

## Discussion

We recently experimentally demonstrated that GBV-B and HCV NS2 exhibit similar membrane topological organizations with three N-terminal transmembrane segments, which we modeled to be also shared by other recently identified mammalian hepaciviruses [22]. In this work, we show that NS2 from hepaciviruses of equine, bat, Old World monkey and rodent origins are cysteine proteases responsible for cleavage at the NS2/NS3 junction (Fig 2), as previously reported by us and others for GBV-B and HCV [22,41,42]. These results thus demonstrate that despite limited aa sequence similarity, NS2 from HCV and related mammalian hepaciviruses share both structural and proteolytic properties.

We further show that the N-terminal hydrophobic domain of NS2 is required for the catalytic activity of BHV, RHV and GBV-B NS2 proteases, but not for that of HCV, NPHV and GHV NS2 (Fig 3B). The characterization of GBV-B NS2 proteolytic activity in a cell-free expression system confirmed the critical role of GBV-B NS2 N-terminal domain interaction with a membrane-mimicking detergent (Fig 3C and 3D). These data indicate that intramolecular interactions of NS2 N-terminal membrane-integrated domain and C-terminal protease domain contribute to the proper folding into a catalytically active domain, at least for a subset of hepaciviruses (BHV, RHV, GBV-B; see NS2 C-terminal homology models in S3 Fig). Interestingly, although not required for NS2 proteolytic activity, interactions between NS2 N- and C-terminal regions have been shown to be critical for HCV NS2 function in particle morphogenesis [22,58]. Accordingly, for HCV and all related mammalian hepaciviruses examined here, interactions between NS2 N-terminal transmembrane region and C-terminal protease domain most likely play a critical role for NS2 proteolytic activity and/or NS2 function in viral particle assembly.

An intriguing feature of HCV NS2 protease is that it is catalytically active as a dimer with two composite active sites, as previously suggested by size-exclusion chromatography analysis of re-folded HCV 2b-J8 NS2 [59] and further substantiated by HCV 1a-H77 NS2 protease domain crystal structure [48]. Importantly, the results presented here indicate that NPHV and GBV-B NS2, like HCV 2a-JFH1 NS2, also form homodimers with two composite active sites, with each catalytic triad composed of the His and Glu residues from one monomer and the Cys residue from the other (Fig 4). Although other virus-encoded or cellular dimeric proteases have been described, the formation of homodimers with two composite active sites is presently a unique feature of hepacivirus NS2 among currently characterized proteases. Indeed, herpes virus, HIV or caspase dimeric proteases form either two active sites with each site contributed by one monomer [60] or only one composite catalytic site [61,62]. The conservation of NS2 particular mode of action between HCV and both closely- (NPHV) and distantly- (GBV-B) related hepaciviruses suggests a critical role for NS2 dimerization in hepacivirus life cycle. An attractive hypothesis is that NS2 protease dimerization could regulate the kinetics of genomic replication initiation, which relies on NS2/NS3 cleavage for the release of nonstructural proteins (NS3-NS5B) comprising the active replicase. Consistent with NS2 dimerization, the rate of HCV NS2/NS3 cleavage is dependent on the concentration of NS2-NS3 precursor until reaching a plateau [59]. As a result, the requirement for minimal amounts of NS2-NS3 precursor could delay hepacivirus genomic replication. Of note, it was shown that rate-limiting NS2-mediated polyprotein cleavage translated into reduced replication competence at least for some HCV isolates, possibly as the result of kinetic differences in membranous replication complex biogenesis [63]. NS2/NS3 cleavage kinetics may also impact the intracellular levels of active NS3/4A protease, modulating the inhibition of innate immune sensing. Indeed, it is known that HCV NS3/4A protease plays a central role in innate immune evasion by cleaving MAVS and Toll-like receptor 3 adaptor protein (TRIF), hence disrupting signaling pathways leading to type I interferon induction [31,32,64]. Interestingly, we and others showed that at least MAVS cleavage is conserved across NS3-4A proteases of various hepaciviruses [29,30,33], suggesting that all hepaciviruses may have evolved similar kinetic regulation of innate immunity evasion strategies, potentially contributing to hepacivirus persistence and pathogenesis.

The current dogma is that efficient NS2-mediated processing at the NS2/NS3 junction is dependent on the stimulation by NS3 N-terminal domain [49]. Conserved hydrophobic residues located at the surface of NS3_N_ were recently shown to play an essential role in this process, as well as in replicase assembly [47]. We demonstrate here that NS3_N_ domains of some but not all mammalian hepaciviruses are able to stimulate HCV NS2 protease (Fig 5) and that this cross-species activation of HCV NS2 protease depends on the conservation or engineering of HCV-like hydrophobic residues in heterologous NS3_N_ surface patches (Fig 6, S3 Fig). In addition, NS3_N_ residues lying at a similar surface location also appeared important for NS3_N_-mediated NS2 protease activation in other nonhuman hepaciviruses (Fig 6). These results strengthen the role of NS3 surface patch for NS3 cofactor activity, indicating that interactions between specific NS3 residues and a yet unidentified region within NS2 could contribute to NS2 protease active site conformation in all hepaciviruses.

In addition to this NS3-dependent proteolytic activity, a major finding of our study is the first-time report of HCV NS2 efficient intrinsic activity in the absence of any NS3 sequence. NS2 substrate specificity was characterized in the context of heterologous fusion of a linker-tag polypeptide to HCV NS2 C-terminus, in the absence of any NS3 sequence. We show that HCV NS2 protease tolerates small aa flexible linkers but not non-flexible linkers or larger P'1 residues (Fig 7, S5 Fig), indicating that the nature of the residues fused immediately downstream of NS2 C-terminus is critical for NS3-independent NS2 proteolytic activity. Of note, some of the permissive 5-aa linkers (GGGGS, GSAGS, GPGGS, APGGS) do not present any sequence homology with NS3 N-terminal residues (APITA). A previous mutagenesis study showed that, although few nonconservative single aa substitutions severely impaired cleavage efficiency, the NS2/NS3 cleavage site of HCV genotype 1a H strain was remarkably resistant to mutation [65]. Our data (Fig 7C and 7D, S5 Fig) support these results and further indicate that NS2 is even more tolerant than expected since NS2 proteolytic activity is resistant to some favorable substitutions of all 5 residues directly fused to its C-terminus (residues P1’ to P5’). In addition, the nature and length of the tag fused downstream of the linker sequence did not impact NS2/linker-tag cleavage efficiency (Fig 7D). This feature contrasts with the stimulatory effect of NS3 N-terminal domain on NS2/NS3 cleavage and with the critical role of NS3 surface patch discussed above. To conciliate these results, one hypothesis would be that NS3 N-terminus may rather have a negative impact on NS2/NS3 cleavage, which can be compensated by the positive effect of selected residues in NS3 protease domain, whose translation and folding may promote NS2 protease stimulation. In this context, the most conserved residue across hepaciviruses on the P' side of NS2/NS3 cleavage site (a proline residue at the P'2 position) did not appear to restrict NS2 protease in a linker-tag fusion context (S5 Fig).

**Fig 11:**
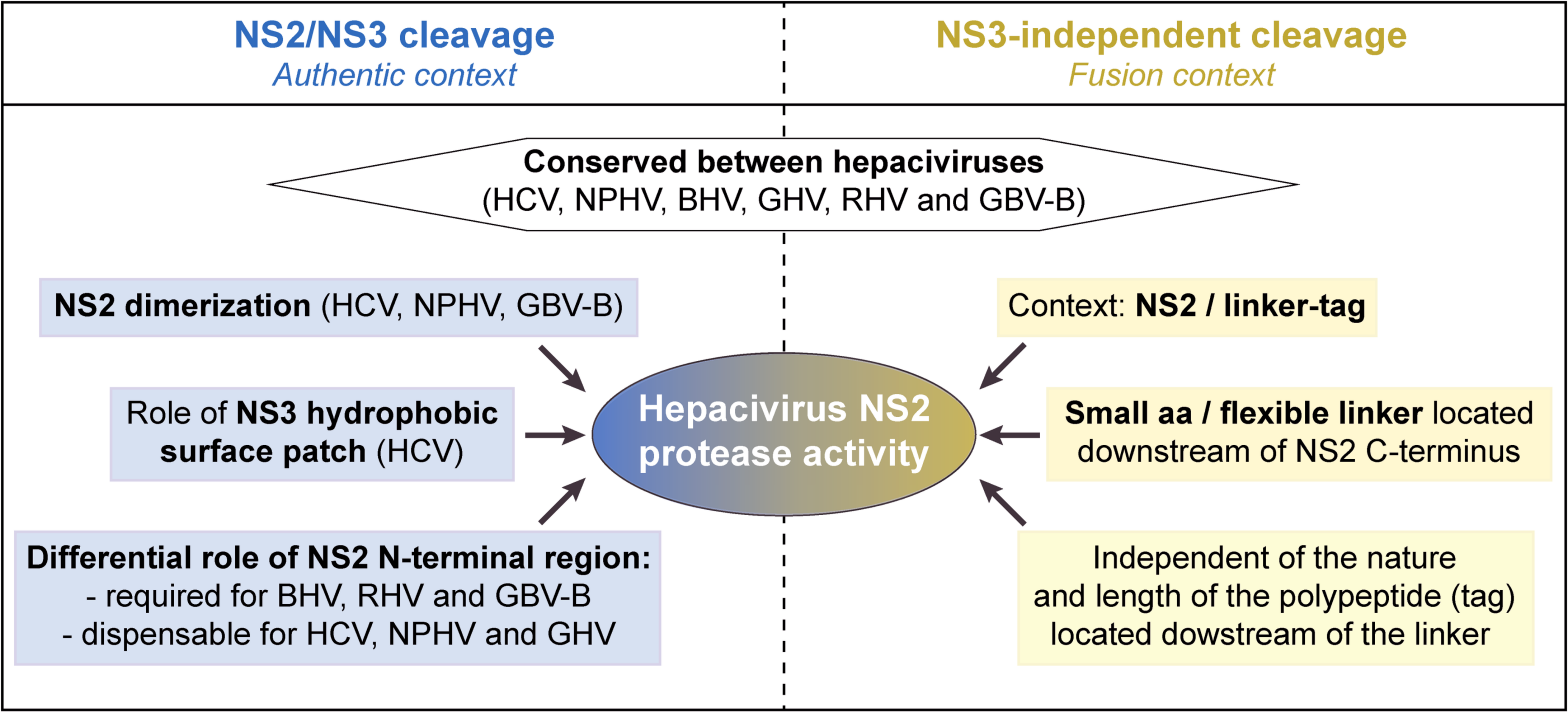
Summary of NS2 protease characteristics across various mammalian hepaciviruses.

Fig 11 summarizes our findings with respect to NS2 protease characteristics across various mammalian hepaciviruses, including (i) its activity responsible for NS2/NS3 cleavage, regulated by NS3 N-terminal domain (particularly NS3 surface patch) and dependent on NS2 dimerization, and (ii) its NS3-independent proteolytic activity. Importantly, the NS3-independent activity described here occurred efficiently in human hepatoma cells supporting virus production and was conserved among all mammalian hepaciviruses studied (Figs 9 and 10). This NS2 intrinsic activity may be exerted through protease dimerization involving precursors with different NS2 C-terminal fusions (Fig 8). It is tempting to speculate that the intrinsic NS2 proteolytic activity may have implications for HCV replication cycle and/or interference of HCV with host regulations. One attractive hypothesis is that HCV NS2 by virtue of its wider substrate specificity may be able to cleave cellular factors. Multifunctionality is a common feature among viral proteins including viral proteases such as HCV NS3 or poliovirus 2A that not only target viral polyproteins but also host cell factors such as MAVS and eIF4G, respectively [31,32,66]. However, HCV NS2 protease inactivation through the mutation of catalytic residues in the context of HCV bicistronic genomes in which an IRES was inserted between NS2 and NS3 sequences had no effect on viral particle production (Fig 10D and S7 Fig), as previously reported [43,44]. This indicates that should they occur, such NS2-mediated additional proteolytic events are not required for the completion of HCV infectious cycle *in vitro*. In addition, although NS2 cleavage of host cell factors is an appealing hypothesis, it should be stressed that in the reported crystal structure of HCV NS2 protease domain [48], NS2 C-terminal residue (Leu 217) remains located within the active site, suggesting that NS2 catalytic site is locked after NS2/NS3 cleavage, which would prevent subsequent proteolytic events. Whether a so far unrecognized alternative post-cleavage conformation co-exists for HCV NS2 following displacement of the C-terminal residues would remain to be shown. Interestingly, a cellular J-domain protein (Jiv), a member of the DNAJ chaperone family, has been shown to act as an activating cofactor of NS2 protease from a related pestivirus, bovine viral diarrhea virus (BVDV). This interaction operates by promoting positioning of NS2 active site and substrate peptide into cleavage competent conformations for *cis*- and *trans*-cleavages [67]. A similar mechanism may exist for hepacivirus NS2 proteases. A high-throughput proteomic approach may allow to address whether NS2 could cleave nonviral substrates in *trans* and seek putative cellular substrates of NS2 protease, as was successfully done for HCV NS3/4A [68].

In conclusion, our results highlight important conserved features among mammalian hepacivirus NS2 proteases, some of which are, to our knowledge described for the first time: (i) hepacivirus NS2 are cysteine proteases acting as dimers forming two composite active sites responsible for the cleavage of the viral polyprotein at the NS2/NS3 junction, and (ii) NS2 proteases from HCV and related hepaciviruses exhibit an NS3-independent efficient proteolytic activity with defined substrate specificity. Our study also underlines a conserved, finely tuned regulation of the viral polyprotein proteolytic cleavage, which may have functional importance in hepacivirus infectious life cycle *in vivo*. Together with other studies pointing to important common characteristics across HCV and recently described hepaciviruses circulating in several mammalian species [11,13,16,18,30,35,37], our data provide further support for the value of these hepaciviruses to be developed as surrogate, immunocompetent animal models of HCV infection [69]. Such models would be instrumental to investigate aspects of HCV-associated pathogenesis and to assess vaccine strategies toward global HCV eradication.

## Materials and methods

### Accession numbers

Viral strains considered in this study and the respective GenBenk accession numbers (in parentheses) are as follows: bat hepacivirus (BHV) PDB-112 (KC796077), BHV PDB-445 (KC796091), BHV PDB-452 (KC796090), BHV PDB-829 (KC796074), GBV-B (AY243572), Guereza hepacivirus (GHV) GHV-1 BWC08 (KC551800), GHV-2 BWC04 (KC551802), HCV 1a-H77 (NC_004102), HCV 1b-Con1 (AJ238799), HCV 2a-JFH1 (AB047639), HCV 2a-J6 (AF177036), HCV 2b-MD2b-1 (AF238486), HCV 3a-CB (AF046866), HCV 3b-TrKj (D49374), HCV 4a-ED43 (NC_009825), HCV 5a-SA13 (AF064490), HCV 6a-EUHK (Y12083), HCV 6b-Th580 (NC_009827), HCV 7a-QC69 (EF108306), nonprimate/equine hepacivirus (NPHV) B10-022 (JQ434004), NPHV G1-073 (JQ434002), NPHV H3-011 (JQ434008), NPHV NZP-1 (JQ434001), rodent hepacivirus (RHV) RHV- 339 (KC815310), RHV NLR07-oct70 (KC411784), RHV RMU10-3382 (KC411777), and RHV SAR-46 (KC411807).

### Plasmids

GBV-B NS2 sequences were amplified from pGBV-B/2, which contains genome-length GBV-B cDNA [4]. HCV 1b-Con1, HCV 2a-JFH1 and 2a-J6 NS2 sequences were amplified from plasmids pCMVNS2-GFP [51] kindly provided by D. Moradpour (Centre Hospitalier Universitaire Vaudois, Lausanne, Switzerland), pJFH1 [70] kindly provided by T. Wakita (National Institute of Infectious Diseases, Tokyo, Japan), and FL-J6/JFH-5'C19Rluc2AUbi [71] kindly provided by C. Rice (The Rockefeller University, New York, NY), respectively. NS2-NS3_N_ coding sequences from BHV PDB-452, GBV-B, GHV-1 BWC08, HCV 2a-JFH1, NPHV H3-011 and RHV NLR07-oct70 (S1 Table) in which the Ser codon of NS3 established / putative catalytic triad was mutated into an Ala codon were sequence-optimized for expression in human cell lines and *de novo* synthesized (GenArt, Life Technologies).

Plasmids pCMV/NS2-NS3_N_-ST and pCMV/NS2-NS3_N_-V5 were modified from pCMV-KEB-GFP [72] by an overlapping-PCR mutagenesis strategy designed to introduce (i) the signal peptide sequence derived from the CD5 cellular gene, (ii) NS2-NS3_N_ native or codon-optimized sequences from the selected hepacivirus, (iii) twin-strep-tag (ST) [WSHPQFEK-(GGGS)_3_-WSHPQFEK] or V5 epitope [GKPIPNPLLGLDST] coding sequence and eliminate enhanced green fluorescent protein (GFP) coding sequence. Plasmids pCMV/NS2^HCV^-NS3_N_^hepaci^-ST were similarly constructed such as to introduce HCV NS2 codon optimized sequence followed by NS3_N_ codon-optimized sequences from the selected hepacivirus at step (ii) and ST at step (iii). Derivatives of plasmids pCMV/NS2^HCV^-NS3_N_^hepaci^-ST and pCMV/NS2-NS3_N_-ST (NPHV, GHV, GBV-B) encoding point mutations of NS3_N_^hepaci^ aa 105, 115/116, and/or 127 were generated by PCR-based site-directed mutagenesis of corresponding codons.

Plasmids pCMV/ΔNS2-NS3_N_-ST were modified from the respective pCMV/NS2-NS3_N_-ST by site-directed mutagenesis in order to delete the following sequences (nucleotide positions within NS2 sequence, using NS2 N-terminal boundaries defined in Fig 1B): (1–279) for HCV and NPHV, (1–264) for BHV and GHV, (1–246) for RHV and (1–258) for GBV-B. Plasmids pCMV/NS2^HCV^±linker-GFP and pCMV/NS2^hepaci^-4GS-GFP were modified from pCMV-KEB-GFP [72] by overlapping-PCR mutagenesis to introduce (i) CD5 signal peptide sequence, (ii) native (GBV-B, HCV 2a JFH1, HCV 2a J6, HCV 1a H77, HCV 1b Con1) or codon-optimized (BHV, GHV, HCV, NPHV and RHV) NS2 sequences and (iii) in appropriate cases, the sequence coding for a linker (SPITA, AP, 4GS [GGGGS], 2x4GS [GGGGSGGGGS], GSAGS, SKSTS, EAAAK or PAPAP) inserted immediately upstream of the GFP coding sequence. Plasmids pCMV/NS2^HCV^-4GS-ST and pCMV/NS2^HCV^-4GS-V5 were generated similarly from pCMV-KEB-GFP to introduce CD5 signal peptide sequence, HCV NS2 codon-optimized sequence, 4GS linker, and ST or V5 tag sequences in place of GFP coding sequence. Plasmids pCMV/NS2^HCV^-linker-GFP, in which the encoded linker is GPGGS, APGGS, NPGGS or SPGGS, were derived from pCMV/NS2^HCV^-4GS-GFP by overlapping, PCR-based site-directed mutagenesis.

To generate pEU plasmids used for cell-free translation in the wheat germ expression system, JFH1 and GBV-B native sequences coding for NS2-NS3_N_-ST or ΔNS2-NS3_N_-ST were PCR amplified and cloned into the pEU-E01-MCS vector (CellFree Sciences).

In order to generate pJad-2EIL3 plasmid, pJad [22,57] was modified such as to insert by overlapping PCR the EMCV IRES cDNA followed by the Firefly luciferase reporter sequence, the FMDV 2A and the ubiquitin coding sequences between NS2 and NS3 coding sequences. Primer-based mutagenesis by overlapping PCR was next used to insert the HA epitope (HAep) sequence [YPYDVPDYA] downstream of HCV NS2 first N-terminal residue (pJad-2EIL3/HAepNS2) and to generate derivatives of pJad-2EIL3/HAepNS2 encoding NS2 C-terminally fused to GFP, ST, or V5 sequences via a 4GS linker (pJad-2EIL3/HAep-NS2-4GS-tag).

For all plasmids described above, derivatives were generated by PCR-based site-directed mutagenesis in order to encode mutated NS2 catalytic triads in which the Cys or His codons were substituted by an Ala codon.

PCR amplified DNA fragments from selected plasmid clones were checked by automated nucleotide sequencing using capillary electrophoresis (Applied Biosystems). Further details of the cloning procedures can be provided upon request.

### Protein sequence analyses

N- and C-terminal boundaries of NS2 and NS3_N_ domains from RHV, BHV, GHV and NPHV were predicted by determining signal peptidase cleavage sites using the SignalP 4.0 server (http://www.cbs.dtu.dk/services/SignalP/) and examining sequence homology with HCV and GBV-B polyproteins. Protein sequence analyses were performed by using the Mobyle portal for bioinformatics analyses (http://mobyle.pasteur.fr/) [73]. Multiple sequence alignments were performed with the T-coffee multiple sequence alignment program [74] using the corresponding web server facility (http://www.tcoffee.org) and aa identity or similarity percentages were calculated according to CLUSTAL W conventions. Phylogenetic trees were constructed using the neighbor joining method under the Jones-Thornton-Taylor model of aa substitution implemented in the MEGA6 program [75] and bootstrap resampling from 2,000 replicates was performed.

### Homology structure models of NS2 proteases and NS3_N_ domains of the various hepaciviruses

Three-dimensional homology models of NS2 and NS3 proteases were constructed by the Swiss-Model automated protein structure homology modeling server [76] (http://www.expasy.org/spdbv/) by using the crystal structures of HCV NS2 protease domain and NS3 as templates (PDB entries: 2HD0 [48] and 1CU1[55], respectively). Figures were generated from structure coordinates by using VMD [77] (http://www.ks.uiuc.edu/Research/vmd/) and rendered with POV-Ray (http://www.povray.org/).

### Cell lines

Human embryonic kidney cells HEK 293T (American Type Culture Collection) were cultured in Dulbecco's modified Eagle's medium (Invitrogen) supplemented with 10% fetal calf serum, 100U/ml penicillin and 100 μg/ml streptomycin (DMEM-10%), at 37°C in a 5% CO_2_ environment. Huh-7.5 human hepatocellular carcinoma cells (Apath, LLC) [78], kindly provided by C.M. Rice, were cultured in DMEM-10% supplemented with nonessential aa and 1mM sodium pyruvate (complete DMEM).

### Antibodies

Rabbit polyclonal antibodies NS2-1519 raised against HCV JFH1 NS2 synthetic peptides (anti-NS2^JFH1^) [44] were kindly provided by R. Bartenschlager. Monoclonal antibodies specific for HCV-JFH1 NS3, GFP (JL-8), V5, ST, and HA were purchased from BioFront, Clontech, Invitrogen, Qiagen, and Sigma-Aldrich, respectively.

### DNA transfection

HEK 293T cells were seeded at a concentration of 2x10^5^ cells per well of 24-well plates and transfected 24 h later with plasmid DNA (0.1–0.8 μg) using *FuGENE 6 Transfection Reagent* (Promega), as recommended by the manufacturer.

### *In vitro* transcription, RNA transfection and monitoring of virus production

Genome-length pJad-2EIL3-based plasmids were linearized with *Mlu*I and served as templates for *in vitro* transcription using *T7 RiboMAX Express Large Scale RNA Production System* (Promega) according to the manufacturer's instructions. Synthetic RNAs were then purified by phenol-chloroform extractions, precipitated with isopropanol, resuspended in RNAse-free water, and stored at -80°C until use for cell transfection. RNA quality and quantity were monitored following 1% agarose gel electrophoresis and measurement of the absorbance at 260 nm. Huh-7.5 cells (2x10^6^ cells) were transfected by electroporation with 5 μg of *in vitro* transcribed genome-length RNA. Cells were electroporated in 4mm-gap width cuvettes by applying one pulse at 240 V, 900 μF (*EasyjecT Plus*, Equibio), then immediately resuspended in complete DMEM and seeded at 2x10^5^ cells per well in 6-well plates. Lysates were prepared using *Reporter Lysis Buffer* (Promega) at 72 h post-transfection from 6-well plates (0.2 mL) or at 72 h post-infection from 12-well plates (0.1 mL) that have been infected with supernatants collected from transfected cells at 72 h post-transfection. Ten-μL aliquots of lysates were assayed for firefly luciferase activity using 50 μL of *Luciferase Assay Reagent* (*Luciferase Assay System*, Promega) and a *Centro LB 960* plate luminometer (Berthold Technologies). Huh-7.5 transfected cell supernatants harvested at 72 h post-transfection were processed for infectivity endpoint dilution titration, essentially as described previously [22] with the following minor modification: 2.5x10^3^ cells were seeded per well of 96-well plates and incubated for 5 days after infection prior to processing for infected foci revelation.

### Wheat germ cell-free protein expression and sample preparation

Wheat germ cell-free protein expression and sample preparation were performed according to the small-scale bilayer method described previously [53,79]. Translation reactions were incubated at 22°C for 16h in 96-well plates. Expression in the presence of detergent was carried out by adding 0.1% MNG-3 in both the reaction mix and the buffered substrate solution. After protein synthesis, the translation reactions were incubated with benzonase on a rolling wheel for 30 min at room temperature (RT), centrifuged at 20,000 g during 30 min at 4°C and the supernatant was purified using *Strep*-Tactin coated magnetic beads (*MagStrep type 2HC* beads, IBA Lifesciences). Purified ST-containing proteins were eluted in 1X Laemmli sample buffer and further diluted in *NuPAGE LDS sample buffer* (Invitrogen) containing 0.71 M 2-mercaptoethanol for SDS-PAGE and immunoblot analysis.

### Immunoblot analysis

RNA or DNA transfected cells were lysed at 48 h and 32–40 h post-transfection, respectively, in *NuPAGE LDS sample buffer* (Invitrogen) containing 0.71 M 2-mercaptoethanol and heated for 10 min at 95°C. Proteins were loaded onto *NuPAGE* 10% or 12% *Bis-Tris gels* (Invitrogen), separated by SDS-PAGE in *MOPS SDS* running buffer (Invitrogen) and transferred to PVDF or nitrocellulose membranes. Membranes were saturated in *DPBS* (Invitrogen) containing 0.1% Tween-20 (PBS-T) and 5% dry skimmed milk at RT for 1 h, prior to incubation for 1 h at RT or overnight at 4°C with one of the following monoclonal antibodies or antisera diluted in PBS-T containing 1% dry skimmed milk: anti-NS2^JFH1^ (1:2000), anti-V5 (1:4000), anti-ST (0.05 μg/ml), anti-GFP (0.125 μg/ml), or anti-HAep (0.25 μg/ml). Following incubation for 1 h at RT with peroxidase-conjugated (chemiluminescence, GE Healthcare) or Dylight 680 or 800nm-conjugated (fluorescence, Thermo Scientific) anti-mouse or anti-rabbit antibodies, proteins were visualized using *ECL Prime Western Blotting Detection Reagent* followed by exposure to *Hyperfilm MP* (GE Healthcare) or an infrared scanner imager (*Odyssey CLx*, Li-Cor), respectively. Quantifications were performed on images acquired with the latter system using Image Studio Lite software.

## Supporting information

S1 Table Accession numbers and protein boundaries of the hepaciviruses used in this study. Boundaries of NS2 and NS3_N_ coding sequences used in constructs are indicated by their nucleotide (nt) positions within respective cDNAs.
(PDF)

S2 Table Predicted molecular masses of hepacivirus polypeptides. Length in amino acids (aa) and predicted molecular masses in kilodaltons (kDa) of NS2-NS3_N_-ST and ΔN(NS2)-NS3_N_-ST precursors and NS3_N_-ST cleaved products are indicated for the different hepaciviruses.
(PDF)

S1 Fig **Phylogenetic analyses of NS3 helicase (A) and NS5B polymerase (B) across hepaciviruses.** NS3 helicase and NS5B amino acid sequences of selected members of the *Hepacivirus* genus were aligned with the T-coffee multiple sequence alignment program [1], using the corresponding web server facility (http://www.tcoffee.org) and phylogenetic trees were constructed using the neighbor joining method under the Jones-Thornton-Taylor model of amino acid substitution implemented in the MEGA6 program [2]. Bootstrap resampling from 2,000 replicates was performed in order to evaluate the reliability of grouping and significant values (>70%) are shown. The trees are drawn to scale with branch lengths proportional to the average number of amino acid substitutions per site, as indicated by the scale bar. The putative N- and C-terminal boundaries of hepacivirus NS3 helicase and NS5B were predicted based on sequence homology with HCV and GBV-B. Colored boxes cluster viruses according to their experimental (GBV-B) or natural (other viruses) hosts, as indicated above boxes. Viral strains considered in this study are highlighted in the respective species-coded colors.
(TIF)

S2 Fig Sequence conservation of NS2 and NS3_N_ regions from selected members of the hepacivirus genus. NS2 and NS3_N_ amino acid sequences of selected members of the *Hepacivirus* genus were aligned with the T-coffee multiple sequence alignment program [1], using the corresponding web server facility (http://www.tcoffee.org). The conservation of NS2 N-terminal (NS2_N_), NS2 C-terminal (NS2_C_) and NS3 N-terminal (NS3_N_) sequences between each indicated hepacivirus and the JFH1 strain of HCV is represented by the percentages of identical (black and grey bars) and similar (white bars) amino acids.
(TIF)

S3 Fig Mammalian hepacivirus NS2 protease and NS3_N_ homology models. Backbones of three-dimensional molecular homology models of NS2 protease domain (left) and NS3_N_ (right) of the indicated viruses are shown in ribbon representations. Homology models were constructed by the Swiss-Model automated protein structure homology modeling server (http://www.expasy.org/spdbv/; [3]) by using the crystal structures of HCV NS2 protease domain and NS3 as templates (PDB entries: 2HD0 [4] and 1CU1 [5], respectively). Figures were generated from structure coordinates by using VMD (http://www.ks.uiuc.edu/Research/vmd/; [6]) and rendered with POV-Ray (http://www.povray.org/). The side-chain atoms of amino acids comprising the catalytic triads of NS2 and NS3_N_ proteases are represented as spheres of the corresponding van der Waals radii and colored red in NS2 and blue in NS3_N_. Models of NS3_N_ provide insight about the location of the hydrophobic surface patch including residues I3, Y105, P115 and L127 in HCV model comparatively to the homologous residues in NS3 models of the other hepaciviruses. These residues are shown in magenta stick representation except residues at position 3 for which only backbone N atoms are shown as van der Waals spheres for clarity. These surface residues are highlighted in the enlargement of NS3_N_ surface patches at the right.
(TIF)

S4 Fig NS2-mediated cleavage of NS2-linker-tag fusion polypeptides. Precursors spanning HCV NS2 C-terminally fused to the indicated linker followed by GFP were expressed in the context of native (wt) or mutated (CA) NS2 catalytic triad. Transfected cell extracts were probed with anti-GFP antibodies. Uncleaved precursors and cleaved products are indicated by closed and open arrowheads, respectively.
(TIF)

S5 Fig Importance of residues at P'1 and P'2 positions of the cleavage site in the linker-tag fusion context. Precursors comprising HCV NS2 C-terminally fused to the indicated linker followed by GFP were expressed in the context of native NS2 catalytic triad. Transfected cell extracts were probed with anti-GFP antibodies. NS2-4GS-GFP polypeptide with an alanine substitution of NS2 catalytic cysteine residue [4GS(CA)] served as a marker for uncleaved precursor. Uncleaved precursors and cleaved products are indicated by closed and open arrowheads, respectively. Dotted lines indicate where lanes originating from the same immunoblot image have been brought together. Quantifications of cleavage rates (% cleaved products over GFP-reactive precursors + cleaved products) were performed on 4 independent extracts subjected to infrared fluorescent immunoblot imaging and are plotted below representative blot images.
(TIF)

S6 Fig Impact of HA epitope N-terminal fusion on NS2-mediated cleavage in the linker-tag fusion context. Precursors spanning HCV NS2 C-terminally fused to 4GS linker followed by GFP and N-terminally fused to a hemagglutinin epitope (HAep-NS2-4GS-GFP) or with no N-terminal fusion (NS2-4GS-GFP) were expressed in the context of native (wt) or mutated (CA) NS2 catalytic triad. Transfected cell extracts were probed with anti-GFP antibodies. Uncleaved precursors and cleaved products are indicated by closed and open arrowheads, respectively. Quantifications of cleavage rates (% cleaved products over GFP-reactive precursors + cleaved products) were performed on 2 independent extracts subjected to infrared fluorescent immunoblot imaging and are plotted below representative blot images.
(TIF)

S7 Fig Impact of NS2 catalytic site inactivation and HAep fusion on viral particle production. Huh7.5 cells were infected with supernatants from cells transfected with the indicated RNAs encoding active NS2 (wt, black bars) or inactive NS2 with a an Ala substitution of the catalytic Cys residue (CA, grey bars) and collected at 72 h post-transfection. Virus production was determined by measuring intracellular luciferase activity in cell extracts prepared at 72 h post-infection. Controls include the parental Jad-2EIL3 RNA, the replication deficient Jad-2EIL3/GAA RNA and the assembly-deficient Jad-2EIL3/ΔEp7 RNA. Means ± standard deviations of 3 independent transfections, each in duplicates are shown.
(TIF)

S1 References (PDF)
