# Supplementary material for: NS2 proteases from hepatitis C virus and related hepaciviruses share composite active sites and previously unrecognized intrinsic proteolytic activities
Source: PLoS Pathog. 2018 Feb 7;14(2):e1006863. doi: 10.1371/journal.ppat.1006863 (PMC5819835; doi:10.1371/journal.ppat.1006863)
Supplement: S1 Table — Boundaries of NS2 and NS3N coding sequences used in constructs are indicated by their nucleotide (nt) positions within respective cDNAs. (PDF) [file ppat.1006863.s001.pdf]

S1 Table. Accession numbers and protein boundaries of the hepaciviruses used in this study.

Virus	Accession number	NS2 coding sequence	$NS3_N$ coding sequence
**BHV PDB-452**	KC796090	nt 2504-3154	nt 3155-3796
**GBV-B**	AY243572	nt 2642-3265	nt 3266-3835
**GHV-1 BWC08**	KC551800	nt 2416-3051	nt 3052-3699
**HCV H77 (1a)**	NC_004102	nt 2769-3419	-
**HCV Con1 (1b)**	AJ238799	nt 2769-3419	-
**HCV J6 (2a)**	AF177036	nt 2780-3430	-
**HCV JFH1 (2a)**	AB047639	nt 2780-3430	nt 3431-4069
**NPHV H3-011**	JQ434008	nt 2758-3408	nt 3409-4047
**RHV NLR07-oct70**	KC411784	nt 2535-3131	nt 3132-3770

Boundaries of NS2 and $NS3_N$ coding sequences used in constructs are indicated by their nucleotide (nt) positions within respective cDNAs.
