# Supplementary material for: NS2 proteases from hepatitis C virus and related hepaciviruses share composite active sites and previously unrecognized intrinsic proteolytic activities
Source: PLoS Pathog. 2018 Feb 7;14(2):e1006863. doi: 10.1371/journal.ppat.1006863 (PMC5819835; doi:10.1371/journal.ppat.1006863)
Supplement: S2 Table — Length in amino acids (aa) and predicted molecular masses in kilodaltons (kDa) of NS2-NS3N-ST and ΔN(NS2)-NS3N-ST precursors and NS3N-ST cleaved products are indicated for the different hepaciviruses. (PDF) [file ppat.1006863.s002.pdf]

S2 Table. Predicted molecular masses of hepacivirus polypeptides

	$NS2\text{-}NS3_N\text{-}ST$		$NS3_N\text{-}ST$		$\Delta N(NS2)\text{-}NS3_N\text{-}ST$	
	Length	Molecular mass	Length	Molecular mass	Length	Molecular mass
**BHV PDB-452**	462 aa	49.9 kDa	245 aa	25.7 kDa	375 aa	39.9 kDa
**GBV-B**	429 aa	46.6 kDa	221 aa	23.0 kDa	345 aa	36.9 kDa
**GHV-1 BWC08**	459 aa	50.3 kDa	247 aa	26.7 kDa	372 aa	40.7 kDa
**HCV JFH1 (2a)**	461 aa	49.0 kDa	244 aa	25.3 kDa	369 aa	38.8 kDa
**NPHV H3-011**	461 aa	50.2 kDa	244 aa	25.2 kDa	369 aa	39.5 kDa
**RHV NLR07-oct70**	443 aa	48.0 kDa	244 aa	25.6 kDa	362 aa	39.0 kDa

Length in amino acids (aa) and predicted molecular masses in kilodaltons (kDa) of $NS2\text{-}NS3_N\text{-}ST$ and $\Delta N(NS2)\text{-}NS3_N\text{-}ST$ precursors and $NS3_N\text{-}ST$ cleaved products are indicated for the different hepaciviruses.
