# Supplementary figures and images for: NS2 proteases from hepatitis C virus and related hepaciviruses share composite active sites and previously unrecognized intrinsic proteolytic activities
Source: PLoS Pathog. 2018 Feb 7;14(2):e1006863. doi: 10.1371/journal.ppat.1006863 (PMC5819835; doi:10.1371/journal.ppat.1006863)

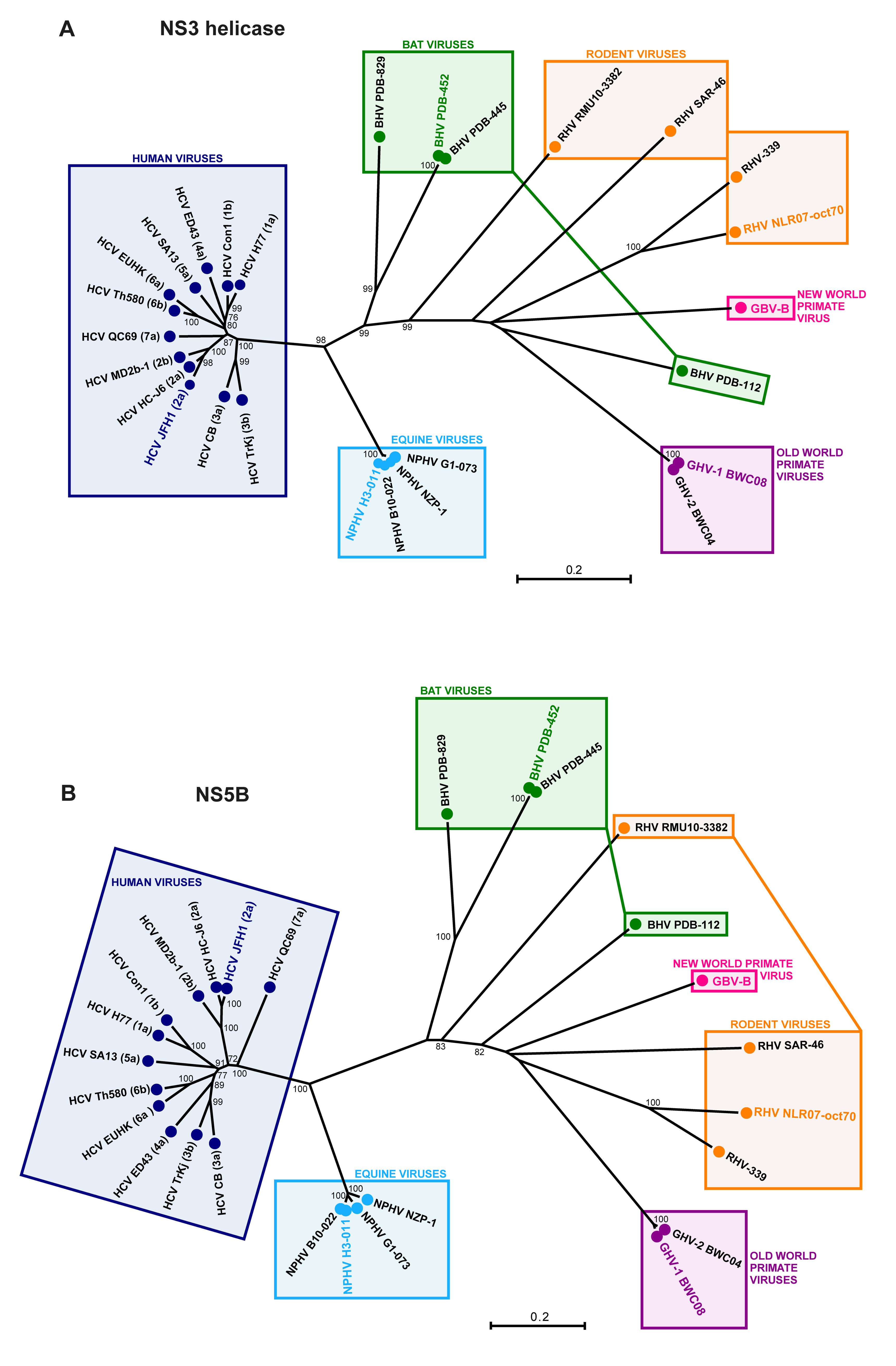

Supplement: S1 Fig — Phylogenetic analyses of NS3 helicase (A) and NS5B polymerase (B) across hepaciviruses. NS3 helicase and NS5B amino acid sequences of selected members of the Hepacivirus genus were aligned with the T-coffee multiple sequence alignment program [1], using the corresponding web server facility (http://www.tcoffee.org) and phylogenetic trees were constructed using the neighbor joining method under the Jones-Thornton-Taylor model of amino acid substitution implemented in the MEGA6 program [2]. Bootstrap resampling from 2,000 replicates was performed in order to evaluate the reliability of grouping and significant values (>70%) are shown. The trees are drawn to scale with branch lengths proportional to the average number of amino acid substitutions per site, as indicated by the scale bar. The putative N- and C-terminal boundaries of hepacivirus NS3 helicase and NS5B were predicted based on sequence homology with HCV and GBV-B. Colored boxes cluster viruses according to their experimental (GBV-B) or natural (other viruses) hosts, as indicated above boxes. Viral strains considered in this study are highlighted in the respective species-coded colors. (TIF) [file ppat.1006863.s003.tif]

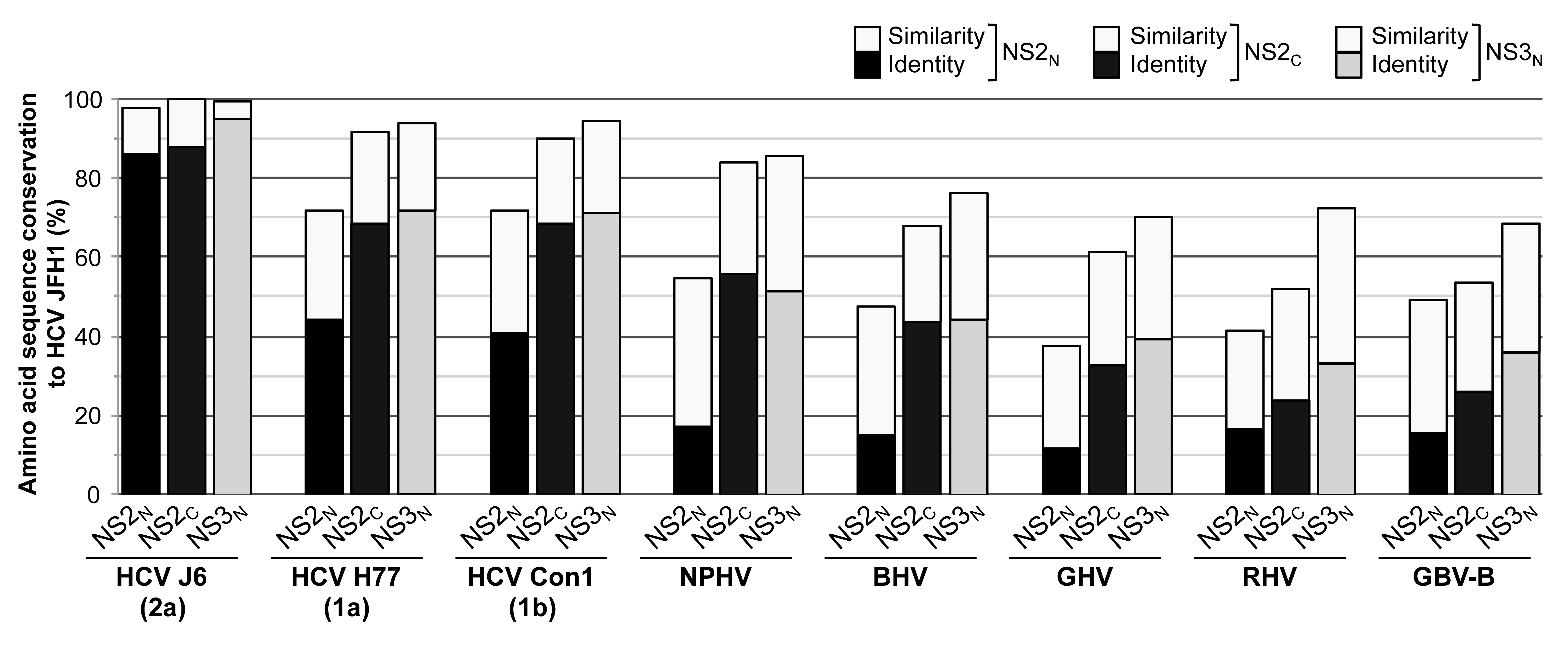

Supplement: S2 Fig — NS2 and NS3N amino acid sequences of selected members of the Hepacivirus genus were aligned with the T-coffee multiple sequence alignment program [1], using the corresponding web server facility (http://www.tcoffee.org). The conservation of NS2 N-terminal (NS2N), NS2 C-terminal (NS2C) and NS3 N-terminal (NS3N) sequences between each indicated hepacivirus and the JFH1 strain of HCV is represented by the percentages of identical (black and grey bars) and similar (white bars) amino acids. (TIF) [file ppat.1006863.s004.tif]

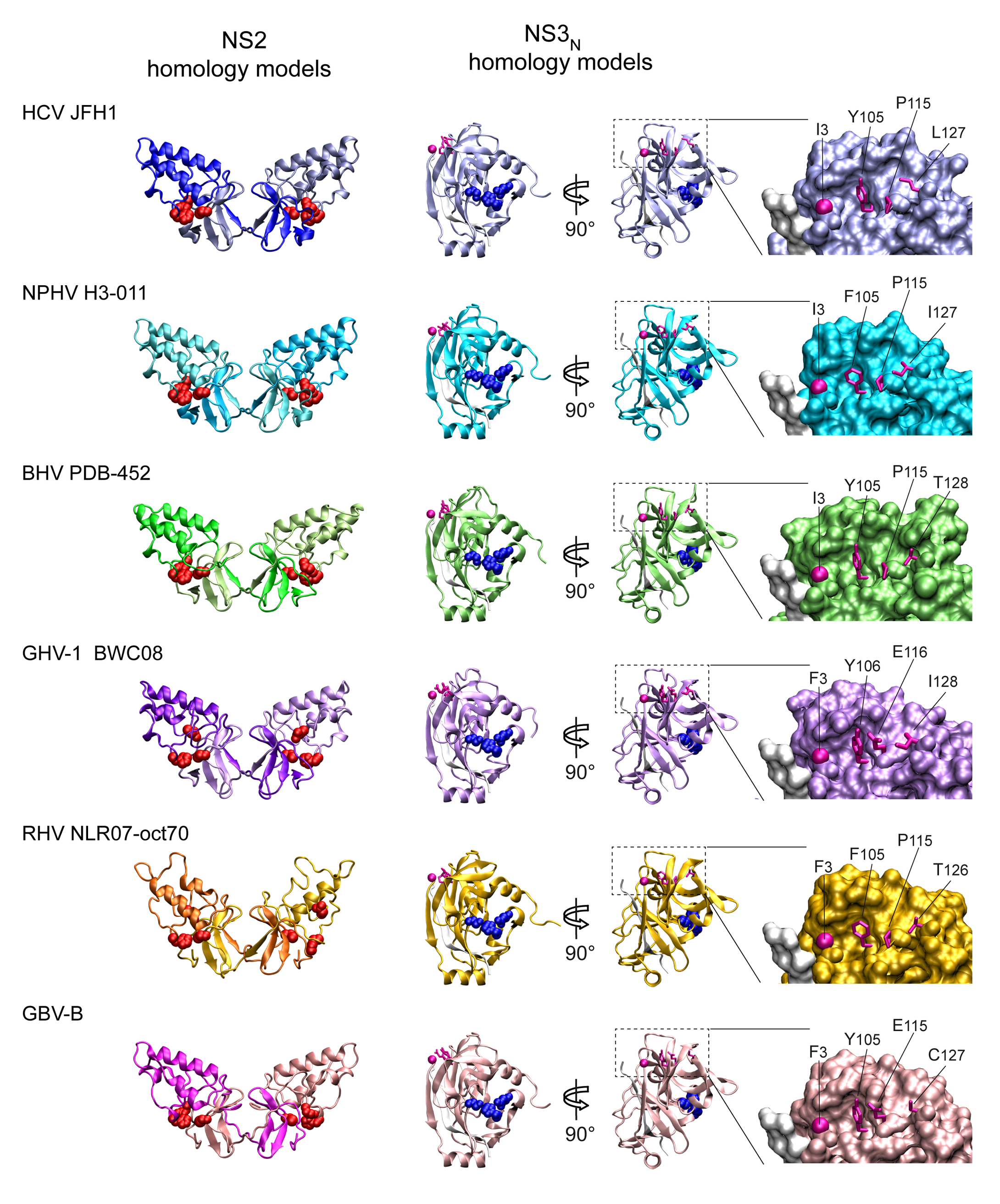

Supplement: S3 Fig — Backbones of three-dimensional molecular homology models of NS2 protease domain (left) and NS3N (right) of the indicated viruses are shown in ribbon representations. Homology models were constructed by the Swiss-Model automated protein structure homology modeling server (http://www.expasy.org/spdbv/; [3]) by using the crystal structures of HCV NS2 protease domain and NS3 as templates (PDB entries: 2HD0 [4] and 1CU1 [5], respectively). Figures were generated from structure coordinates by using VMD (http://www.ks.uiuc.edu/Research/vmd/; [6]) and rendered with POV-Ray (http://www.povray.org/). The side-chain atoms of amino acids comprising the catalytic triads of NS2 and NS3N proteases are represented as spheres of the corresponding van der Waals radii and colored red in NS2 and blue in NS3N. Models of NS3N provide insight about the location of the hydrophobic surface patch including residues I3, Y105, P115 and L127 in HCV model comparatively to the homologous residues in NS3 models of the other hepaciviruses. These residues are shown in magenta stick representation except residues at position 3 for which only backbone N atoms are shown as van der Waals spheres for clarity. These surface residues are highlighted in the enlargement of NS3N surface patches at the right. (TIF) [file ppat.1006863.s005.tif]

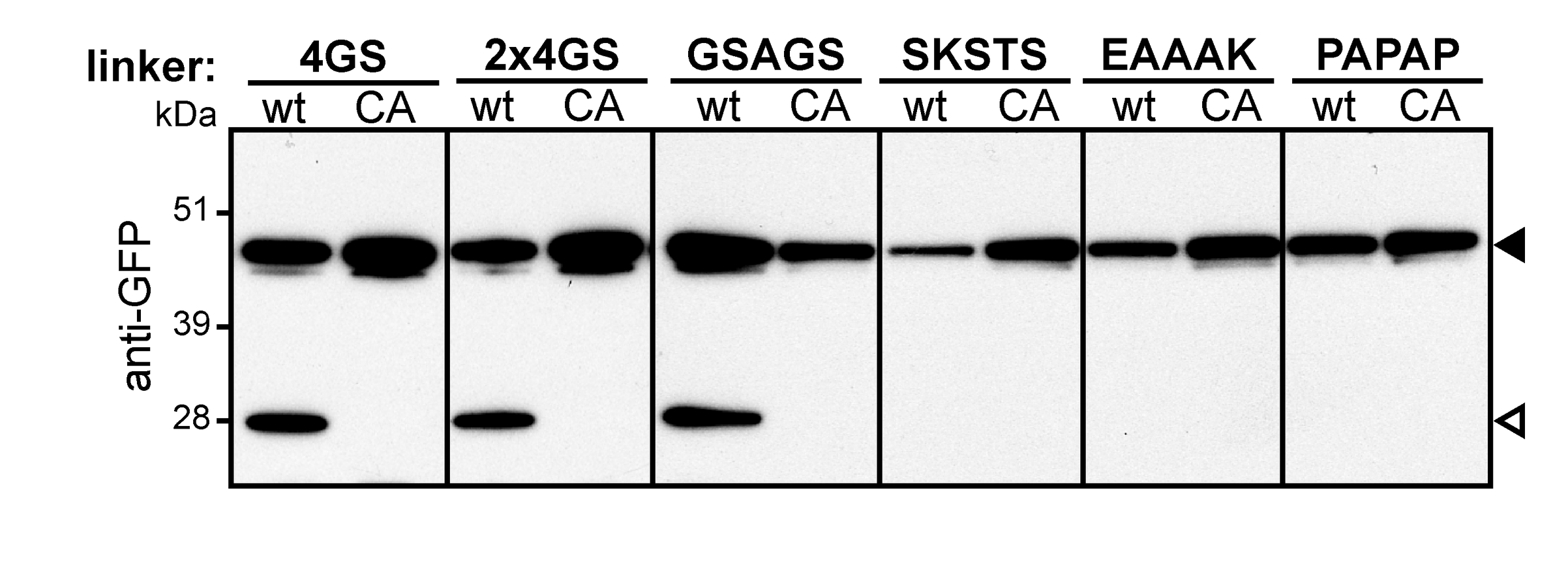

Supplement: S4 Fig — Precursors spanning HCV NS2 C-terminally fused to the indicated linker followed by GFP were expressed in the context of native (wt) or mutated (CA) NS2 catalytic triad. Transfected cell extracts were probed with anti-GFP antibodies. Uncleaved precursors and cleaved products are indicated by closed and open arrowheads, respectively. (TIF) [file ppat.1006863.s006.tif]

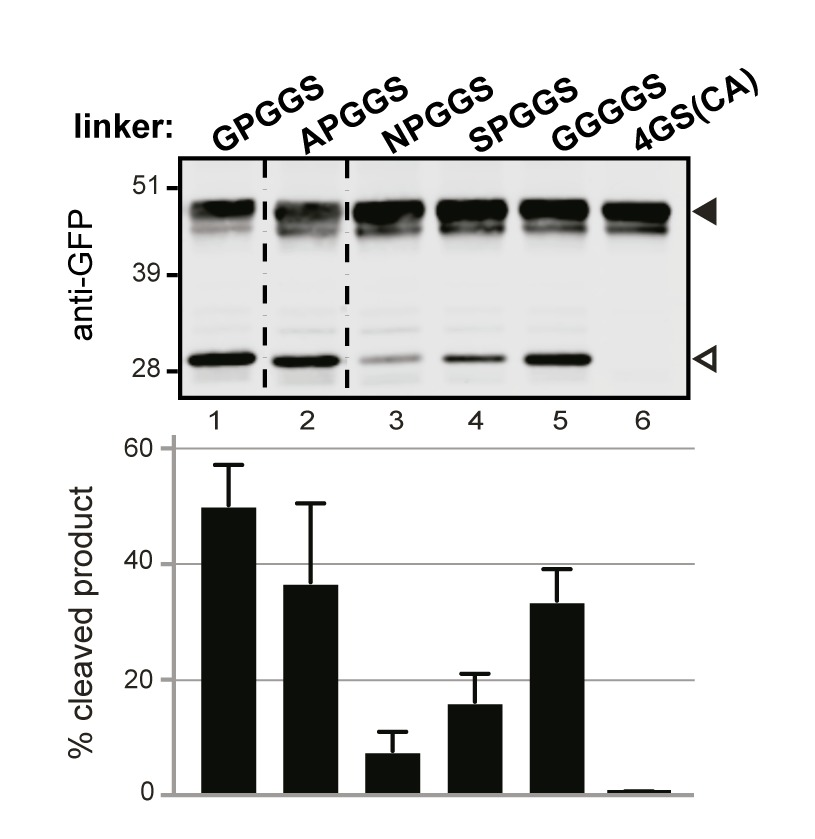

Supplement: S5 Fig — Precursors comprising HCV NS2 C-terminally fused to the indicated linker followed by GFP were expressed in the context of native NS2 catalytic triad. Transfected cell extracts were probed with anti-GFP antibodies. NS2-4GS-GFP polypeptide with an alanine substitution of NS2 catalytic cysteine residue [4GS(CA)] served as a marker for uncleaved precursor. Uncleaved precursors and cleaved products are indicated by closed and open arrowheads, respectively. Dotted lines indicate where lanes originating from the same immunoblot image have been brought together. Quantifications of cleavage rates (% cleaved products over GFP-reactive precursors + cleaved products) were performed on 4 independent extracts subjected to infrared fluorescent immunoblot imaging and are plotted below representative blot images. (TIF) [file ppat.1006863.s007.tif]

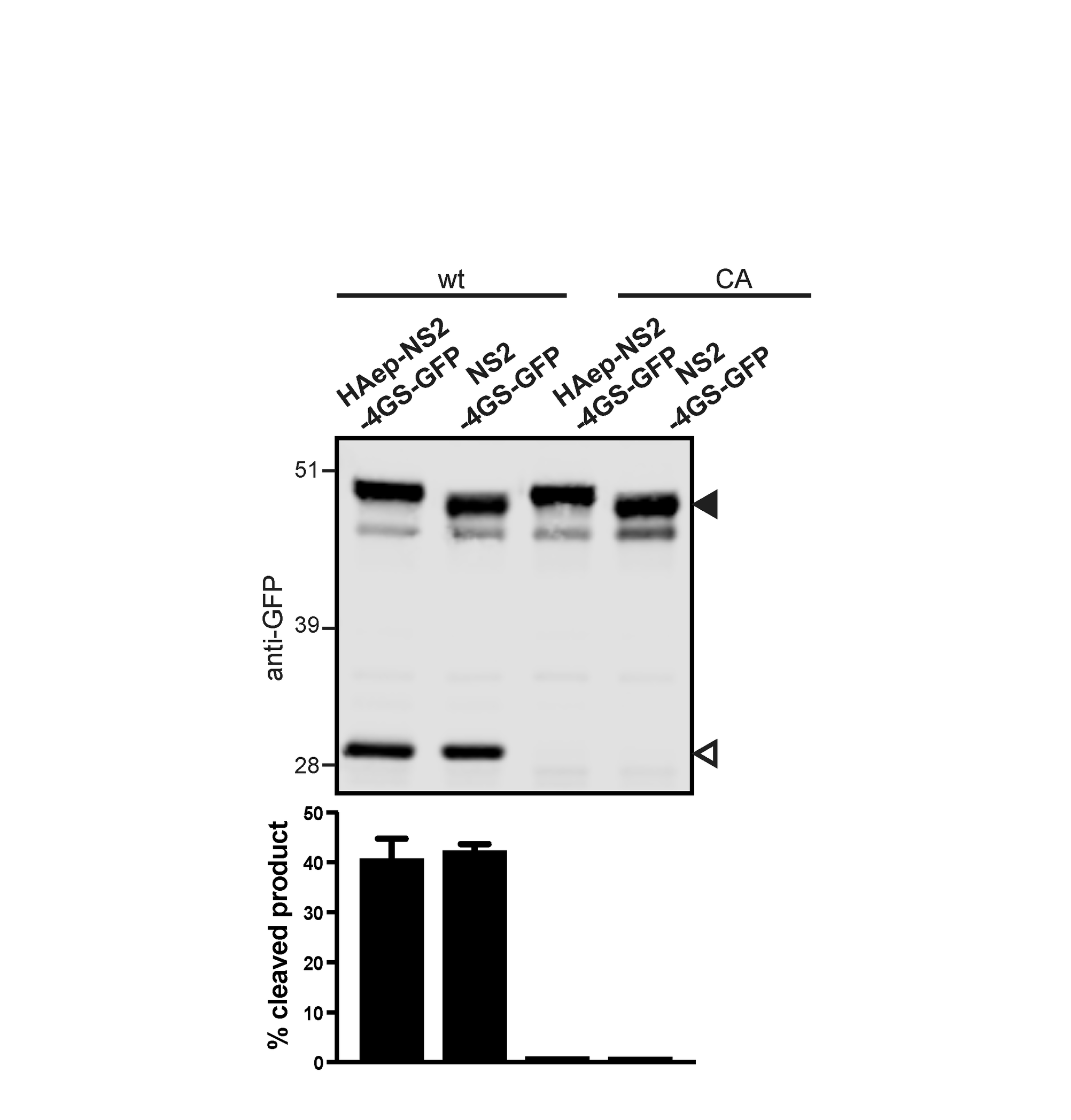

Supplement: S6 Fig — Precursors spanning HCV NS2 C-terminally fused to 4GS linker followed by GFP and N-terminally fused to a hemagglutinin epitope (HAep-NS2-4GS-GFP) or with no N-terminal fusion (NS2-4GS-GFP) were expressed in the context of native (wt) or mutated (CA) NS2 catalytic triad. Transfected cell extracts were probed with anti-GFP antibodies. Uncleaved precursors and cleaved products are indicated by closed and open arrowheads, respectively. Quantifications of cleavage rates (% cleaved products over GFP-reactive precursors + cleaved products) were performed on 2 independent extracts subjected to infrared fluorescent immunoblot imaging and are plotted below representative blot images. (TIF) [file ppat.1006863.s008.tif]

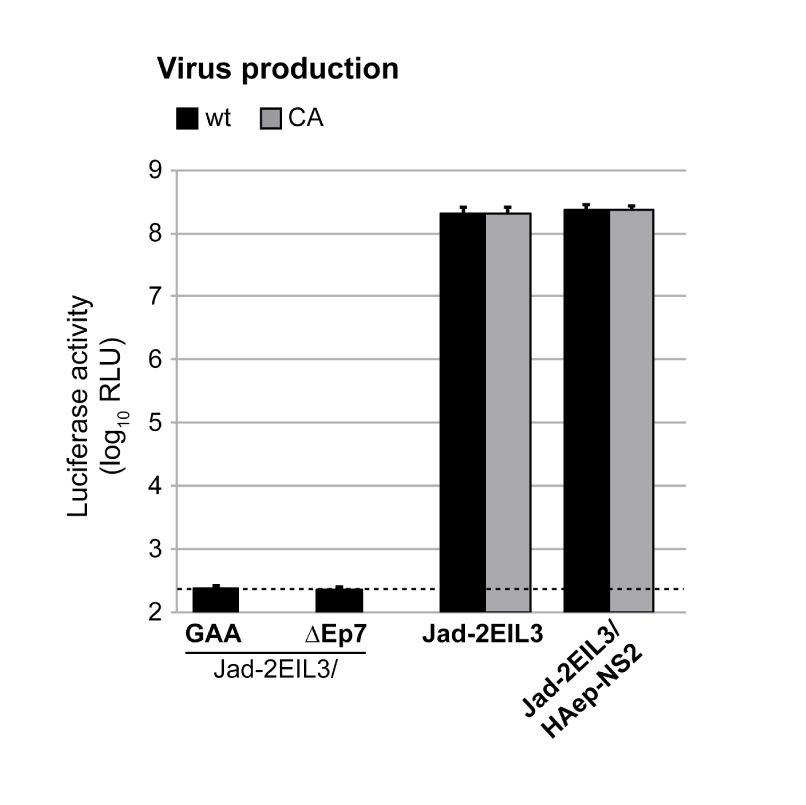

Supplement: S7 Fig — Huh7.5 cells were infected with supernatants from cells transfected with the indicated RNAs encoding active NS2 (wt, black bars) or inactive NS2 with a an Ala substitution of the catalytic Cys residue (CA, grey bars) and collected at 72 h post-transfection. Virus production was determined by measuring intracellular luciferase activity in cell extracts prepared at 72 h post-infection. Controls include the parental Jad-2EIL3 RNA, the replication deficient Jad-2EIL3/GAA RNA and the assembly-deficient Jad-2EIL3/ΔEp7 RNA. Means ± standard deviations of 3 independent transfections, each in duplicates are shown. (TIF) [file ppat.1006863.s009.tif]
