# Supplementary material for: NS2 proteases from hepatitis C virus and related hepaciviruses share composite active sites and previously unrecognized intrinsic proteolytic activities
Source: PLoS Pathog. 2018 Feb 7;14(2):e1006863. doi: 10.1371/journal.ppat.1006863 (PMC5819835; doi:10.1371/journal.ppat.1006863)
Supplement: S1 References — (PDF) [file ppat.1006863.s010.pdf]

Supporting Information References

1. Notredame C, Higgins DG, Heringa J. T-Coffee: A novel method for fast and accurate multiple sequence alignment. J Mol Biol. 2000;302: 205-217.
2. Tamura K, Stecher G, Peterson D, Filipski A, Kumar S. MEGA6: Molecular Evolutionary Genetics Analysis version 6.0. Mol Biol Evol. 2013;30: 2725-2729.
3. Biasini M, Bienert S, Waterhouse A, Arnold K, Studer G, Schmidt T, et al. SWISS-MODEL: modelling protein tertiary and quaternary structure using evolutionary information. Nucleic Acids Res. 2014;42: W252-258.
4. Lorenz IC, Marcotrigiano J, Dentzer TG, Rice CM. Structure of the catalytic domain of the hepatitis C virus NS2-3 protease. Nature. 2006;442: 831-835.
5. Yao N, Reichert P, Taremi SS, Prosise WW, Weber PC. Molecular views of viral polyprotein processing revealed by the crystal structure of the hepatitis C virus bifunctional protease-helicase. Structure. 1999;7: 1353-1363.
6. Humphrey W, Dalke A, Schulten K. VMD: visual molecular dynamics. J Mol Graph. 1996;14: 33-38, 27-38.
